# Fusaric acid-mediated *S*-glutathionylation of MaAKT1 channel confers the virulence of *Foc* TR4 to banana

**DOI:** 10.1371/journal.ppat.1013066

**Published:** 2025-04-09

**Authors:** Jun Zhang, Siwen Liu, Wenlong Yang, Yanling Xie, Chuange Shao, Zhi-Ren Zhang, Chunyu Li, Xiaoqiang Yao

**Affiliations:** 1 Department of Cardiology, The First Affiliated Hospital of Harbin Medical University, NHC Key Laboratory of Cell Transplantation, Key Laboratories of Education Ministry for Myocardial Ischemia Mechanism and Treatment, Harbin, China; 2 Institute of Fruit Tree Research, Guangdong Academy of Agricultural Sciences, Key Laboratory of South Subtropical Fruit Biology and Genetic Research Utilization, Ministry of Agriculture and Rural Affairs, Guangdong Provincial Key Laboratory of Tropical and Subtropical Fruit Tree Research, Guangzhou, China; 3 School of Biomedical Sciences, Li Ka Shing Institute of Health Science, the Chinese University of Hong Kong, Hong Kong, China; 4 Guangdong Laboratory for Lingnan Modern Agriculture, Guangzhou, China; 5 Maoming Branch, Guangdong Laboratory for Lingnan Modern Agriculture, Maoming, China; 6 Centre for Cell & Developmental Biology, School of Life Sciences, The Chinese University of Hong Kong, Hong Kong, China; Purdue University, UNITED STATES OF AMERICA

## Abstract

Our previous studies have demonstrated that the phytotoxin fusaric acid (FSA), secreted by several *Fusarium* species, acts as a key factor in the development of plant diseases; however, the underlying mechanism remains unknown. In this study, we showed that the symptoms of Fusarium wilt in banana seedlings closely resembled those observed in plants grown under potassium (K^+^) deficiency conditions. Mechanistically, we found that FSA induces the accumulation of intracellular reactive oxygen species (ROS), which in turn inhibits banana K^+^ in banana roots. This inhibition occurs via *S*-glutathionylation of the banana AKT1 (MaAKT1) channel, leading to reduced K^+^ influx and reduced K^+^ content in banana roots. Through mutagenesis, electrophysiological studies, immunofluorescence staining, and co-immunoprecipitation experiment, we demonstrated that mutation of Cys202, a highly conserved site in the transmembrane segment 5 of MaAKT1, diminished the biochemical interaction of glutathione (GSH) and the channel induced by FSA, and alleviated *Fusarium oxysporum* f. sp. *cubense* tropical race 4 (*Foc* TR4) and FSA-induced yellowing symptom. The evolutionarily conserved function of this site for *S*-glutathionylation was also observed in *Arabidopsis* AKT1 (AtAKT1) channel, as mutation of its homologue site in AtAKT1 similarly reduced the GSH-AtAKT1 interaction under FSA stress. Collectively, our results suggest that FSA contributes to disease progression by decreasing K^+^ absorption through *S*-glutathionylation of MaAKT1 channel at the conserved Cys202 residue. These findings uncover a previously unrecognized role of FSA in regulating K^+^ homeostasis in bananas, and provide a foundation for future strategies to treat Fusarium wilt and increase banana production by targeting the conserved *S*-glutathionylation site in MaAKT1 channel.

## Author summary

Fusaric acid (FSA) is a secondary metabolite commonly produced by *Fusarium* strains during plant infection. Although FSA has been identified as a virulence factor in several pathosystems, its mechanism of action has not been fully understood. In this study, we characterize a novel mode of action for FSA and link it to the symptoms observed in plants treated with this phytotoxin. We show that FSA confers the virulence of *Fusarium oxysporum* f. sp. *cubense* tropical race 4 (*Foc* TR4) by inhibiting the MaAKT1 K^+^ uptake channel, thereby suppressing K^+^ absorption and reducing K^+^ content in banana roots. Additionally, we demonstrate that FSA increases intracellular ROS levels, which subsequently inhibits MaAKT1 channel by *S*-glutathionylation of the channel at an evolutionarily conserved site. This study provides new insights into the molecular mechanisms by which pathogens perturb plant nutrition pathways and contributes to our understanding of *Fusarium* pathogenesis in various crop systems.

## Introduction

Banana (*Musa* spp.) is one of the most widely cultivated fruits in tropical and subtropical regions, especially in Southeast Asia, Africa, and Latin America, and ranks among the top ten food crops in the world [1]. However, banana production is seriously threatened by Fusarium wilt of banana, also known as Panama disease, caused by the infection of *Fusarium oxysporum* f. sp. *cubense* (*Foc*). In recent years, a substrain *Foc* tropical race 4 (*Foc* TR4) has been causing the most damage. When the banana tree suffers from the disease, it develops chlorosis symptoms, which progresses from lower to upper leaves and finally to the whole plant [2]. Except for a few resistant variants, no effective control measures are currently available. *Foc* employs several fungal mechanisms to manipulate host immunity and enhance its virulence, including the secondary metabolites fusaric acid (FSA) [3–5], beauvericin (BEA) [5], and effector proteins encoded by the *Secreted in Xylem* (SIX) genes [6,7]. Among these, FSA is a major virulent factor secreted by *Foc* TR4 to mediate Fusarium wilt of banana.

FSA is a common contaminant of maize and grains, produced by *Foc* and other members of the *F. oxysporum* species complex [8]. FSA production is influenced by the virulence of the fungal strain and the susceptibility of the host plants [9]. Due to the pathogenic function of FSA in several *Fusarium* species, the genes involved in FSA biosynthetic and its regulatory processes have been extensively studied [10–12]. FSA production is tightly regulated by genes in the fusaric acid biosynthetic (FUB) gene cluster, which includes *FUB1-12* in *Fusarium* species across the entire genus [13]. Additionally, two members of the fungal-specific velvet complex, Vel1 and Lae, are involved in this regulation [14]. The significance of these 12 genes varies, as deletion mutants of each *FUB* gene affect FSA production differently. Among them, *FUB1* plays a key role in the *FUB* gene cluster. Targeted deletion of *Foc FUB1* was reported to completely abolish FSA production in *F. oxysporum* [11,15]. Deficiency in *FUB3, FUB6,* or *FUB8* also abolished FSA production in *F. oxysporum*, but only partially reduces FSA production in corresponding *F. verticillioides* mutants [11]. Interestingly, *FUB10* functions as a transcription factor that regulates the transcription of other FUB genes. Deletion of *FUB10* has been shown to abolish FSA production in *F. verticillioides, F. fujikuroi,* and *F. oxysporum* [10,11]. Deletion of *FUB12* results in incomplete loss of FSA production in *F. verticillioides* and *F. fujikuroi* [10,11]. Our previous study demonstrated that inactivation of *FUB1*, *FUB3*, *FUB4* and *FUB10* in *Foc* TR4 reduced FSA production and resulted in decreased disease symptoms and reduced fungal biomass in the host banana plants [12].

Structurally, FSA contains a nitrogen atom in the pyridine ring and an adjacent oxygen atom in the carboxylic group, making it a powerful divalent cation (including iron, zinc, copper, nickel, and lead) chelating agent [16]. By chelating essential metal ions, FSA disrupts the structural integrity and function of metalloproteins [17]. Known for its high phytotoxicity, FSA contributes to the virulence of plant pathogenic strains of *Fusarium* spp. and the development of the wilting disease in several crops, including tomatoes, bananas, maize, potatoes, cotton, and cape gooseberry. In tobacco suspension cells, low concentration of FSA causes cytoplasmic shrinkage, chromatin condensation, DNA fragmentation, membrane plasmolysis, and small vacuoles formation [18]. In potatoes, FSA causes rapid reversible accumulation of H_2_O_2_ in cells, promotes lipid peroxidation, and increases antioxidant enzymes activity [19]. In maize, FSA inhibited seed germination and seedlings growth by inducing respiratory inhibition, electrolyte leakage, and cytological alteration [20]. FSA caused a dose-dependent inhibition of cell growth and an oxidative burst in *Arabidopsis* [21] and tomatoes [9]. In tomatoes, FSA mediates the assembly of disease-suppressive rhizosphere microbiota by inducing shifts in plant root exudates [22]. For bananas, FSA acts as a key molecule that instigates *Foc* TR4 invasion, causing necrotic spots on leaves, wilting, and shriveling of stems and petioles [12,15]. FSA is presented in all organs in diseased plants, and its concentration was positively correlated with the prevalence and severity of the disease symptoms [3]. Moreover, the pathogenic effects of *Foc* TR4 on banana leaf can be replicated by pure FSA [3]. Despite these findings, the underlying mechanism by which FSA aggravates the infection remains unclear.

Potassium (K^+^) is an essential macronutrient for plant metabolism, growth, and stress adaptation. Its deficiency impairs growth and photosynthesis, causing symptoms such as brown scorching and curling of leaf tips and interveinal chlorosis [23]. Plants grown in K^+^-deficient conditions are more susceptible to abiotic and biotic stresses, such as drought, cold, salinity, and fungal diseases [24]. For example, leaf K^+^ content in apples is negatively correlated with the incidence and severity of Valsa canker disease caused by *Valsa mali* [25]. Conversely, increased K^+^ content enhances fungal resistance in rice, while decreased K^+^ levels reduces immunity [26]. In banana plantlets, K^+^ influences the incidence of Fusarium wilt with K^+^ deficiency being associated with a higher disease incidence, while K^+^ replenishing reduces it [27]. However, it is unknown whether *Foc* TR4 infection and FSA affect K^+^ homeostasis in banana plantlets.

The K^+^ concentration in plant cytosol is typically around 100 mM, while the K^+^ concentration in the soil solution ranges from 100 μM to 1 mM, often lower [28]. To maintain cellular K⁺ levels, plants uptake K⁺ from the soil via K⁺ channels and transporters in the roots. Among these, the HAK5-like transporter and the Shaker-like inward-rectifier AKT1 channel are the primary K⁺ uptake mechanisms [29]. HAK5, which mediates K⁺ uptake at low external concentrations (1 μM to 200 μM), has a limited capacity for K⁺ absorption. In contrast, AKT1 is responsible for the majority of K⁺ uptake across a broad range of environmental K⁺ concentrations (10 μM to 10 mM) [30,31]. AKT1 is expressed in the root epidermis, endodermis, and cortex, where it mediates K^+^ acquisition [32,33].

In the present study, we employed electrophysiological, molecular biological, and transgenic methods to investigate how *Foc* TR4 and FSA disrupt host K⁺ homeostasis to exacerbate disease. Our results showed that *Foc* TR4-infected or FSA-treated banana plantlets develop similar pathological symptoms to K^+^ deficiency. In whole-cell patch-clamp experiments, FSA inhibited the activity of the banana AKT1 channel (MaAKT1). FSA treatment also reduced K^+^ content in plantlets and induced net K^+^ efflux (loss) from roots. Mechanistically, FSA treatment increased reactive oxygen species (ROS) level and promoted *S*-glutathionylation modification of MaAKT1 at Cys202, a highly conserved site among AKT1 channels in different plant species, inhibiting AKT1-mediated K^+^ uptake and leading to net K^+^ loss, thereby aggravating *Foc* TR4 infection. Our study provides new insights into the mechanistic basis of FSA-induced pathogenesis in banana plants.

## Results

### K^+^ deficiency as a symptom of *Foc* TR4 infection and FSA treatment

**Fig 1:**
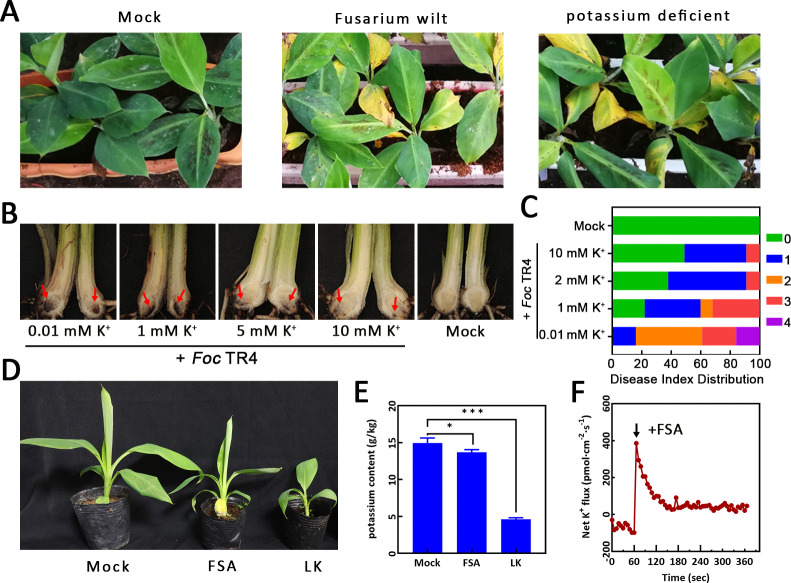
Symptoms of potassium (K^+^) deficiency in banana plantlets infected with *Fusarium oxysporum* f. sp. *cubense* tropical Race 4 (*Foc* TR4) or treated with FSA. (**A**) Comparison of symptoms between Fusarium wilt and K^+^-deficient banana plantlets. (**B**) Disease phenotype and (**C**) Disease index (DI) distribution in banana plantlets inoculated with *Foc* TR4 under varying K^+^ conditions. (**D**) Comparison of symptoms and (**E**) K^+^ content between FSA-treated and K^+^-deficient banana plantlets. Data are presented as means ± SE (n = 3). Student’s t-test, *p < 0.05; ***p < 0.001. (**F**) Effect of FSA on net K^+^ fluxes in the root tip zone of banana (indicated by the arrow).

Potassium (K^+^) deficiency is known to increase plant susceptibility to fungal infections, including Fusarium wilt [27]. To investigate whether *Foc* TR4 infection affects K^+^ homeostasis in banana plantlets, we compared the phenotypic responses of plantlets infected with *Foc* TR4 and those treated with a K^+^-free solution. Thirty days post-treatment (dpt), both groups exhibited similar symptoms, with older leaves turning yellow (Fig 1A).

To explore the impact of varying K^+^ levels on banana resistance to *Foc* TR4, we generated 4 different K^+^ levels in Hoagland solution: 0.01 mM K^+^ (low K^+^/LK), 1 mM K^+^, 5 mM K^+^, and 10 mM K^+^ (high K^+^/HK). Thirty days post inoculation (dpi) of *Foc* TR4, necrosis in the corms decreased with increasing K^+^ concentration (Fig 1B), and the disease index (DI) was significantly lower under high K^+^ conditions compared to that under low K^+^ conditions (Fig 1C).

Our previous studies have shown that FSA acts as a pioneer molecule, instigating the invasion of banana by *Foc* TR4 [12]. To further investigate whether FSA induces K^+^ deficiency symptoms, we treated banana plantlets with FSA. The results demonstrated that FSA-treated plants exhibited similar symptoms to those under K^+^ deficiency conditions (Fig 1D). We also measured the K^+^ content in the roots, revealing that K^+^ levels was significantly lower in FSA- and low K^+^-treated plantlets compared to the mock-treated controls (Fig 1E). Additionally, FSA treatment induced a significant net K^+^ efflux in the root elongation zones of banana roots (Fig 1F).

### MaAKT1 mediates inward rectifier K^+^ currents in HEK-293 cells

To investigate the molecular mechanisms underlying disrupted K^+^ homeostasis in banana roots during *Foc* TR4 infection, we analyzed publicly available transcriptome data of banana roots inoculated with *Foc* TR4 (NCBI accession No. PRJNA1113144). Among the upregulated K^+^-related genes, *AKT1* (designated as *MaAKT1*) was notably induced during *Foc* infection (S1A Fig). We then examined the expression of *MaAKT1* in banana plantlets treated with FSA. RT-qPCR analysis showed that MaAKT1 expression increased progressively during the first 30 minutes of FSA treatment (S1B Fig).

To explore whether MaAKT1 belongs to the Shaker K^+^ channel family members, we conducted sequence alignment of MaAKT1 to its homologues from other plant species. The results showed that MaAKT1 shares significant similarity with Shaker K^+^ channels from other plant species, such as AtAKT1 (64.11%), ZmAKT1 (68.3%), OsAKT1 (67.77%), TaAKT1 (68.4%), StAKT1 (65.8%), GmAKT1 (65%), SlAKT1 (67.4%) (S2 Fig). Phylogenetic analysis clustered the K^+^ channels from monocots and dicots separately (S3 Fig). The MaAKT1 contains a typical TxxTxGYG motif in the putative P-loop domain, a signature of K^+^-selective channels [34], suggesting its high ionic selectivity for K^+^.

**Fig 2:**
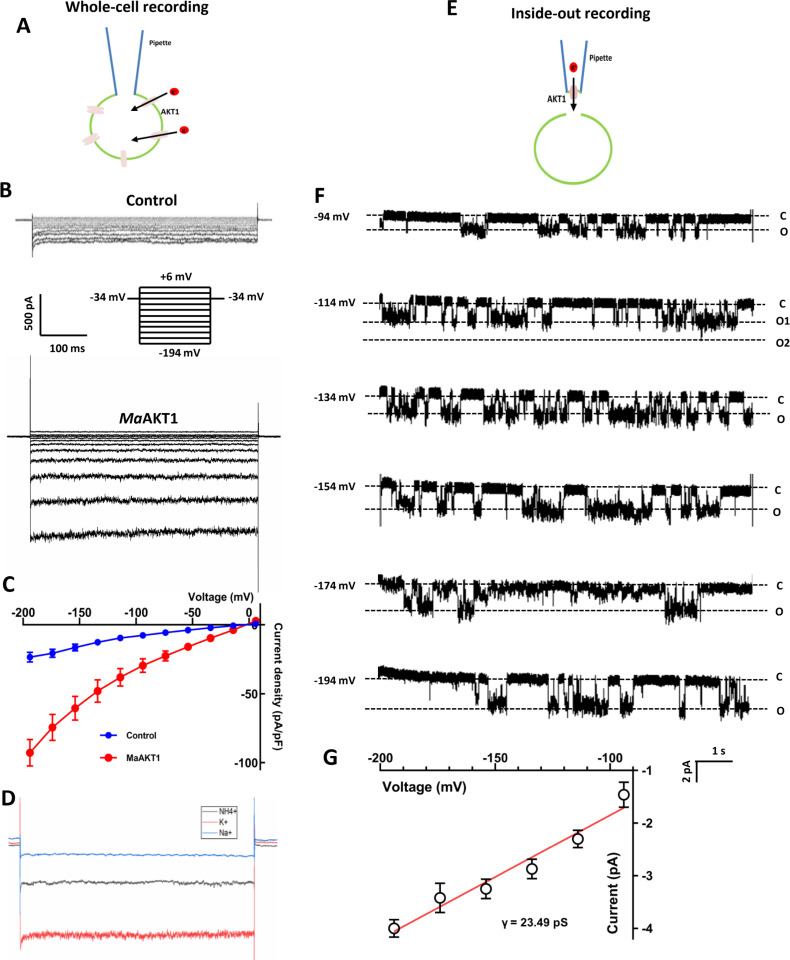
Electrical properties of MaAKT1 channels expressed in HEK-293 cells. (**A**) Diagram illustrating whole-cell recording from HEK-293 cells overexpressing MaAKT1 channels. (**B-C**) Representative whole-cell current traces from control (pcDNA6 vector-transfected) and MaAKT1-transfected HEK-293 cells, recorded with the standard bath and pipette solutions. The insert shows voltage pulses from +6 mV to −194 mV in 20 mV decrements for 500 ms, with a holding potential of −34 mV. (**C**) Summarized current density of background current in HEK-293 cells (n = 9) and MaAKT1-mediated inward rectifying K^+^ current (n = 25). (**D**) Representative current traces recorded at −194 mV in the presence of 100 mM K^+^ (red), NH_4_^+^ (black), and Na^+^ (blue) in the bath solution. **(E)** Diagram illustrating inside-out recording from tiny patches excised from cell membrane of HEK-293 cells overexpressing MaAKT1 channels. (**F**) Representative single-channel current traces from inside-out patches excised from HEK-293 cells, showing inward currents at hyperpolarizing voltages. Pipette and bath solutions contained 150 mM and 100 mM K-gluconate, respectively (**G**) Single-channel conductance (γ) determined by linear regression (red solid line) (n = 4–11 for each data point).

To determine whether MaAKT1 mediates inward K^+^ current, we decided to transient overexpress it in protoplasts isolated from banana embryogenic cell suspensions (ECSs) and conduct patch-clamp recording. However, due to the low transfection efficiency of the protoplast, HEK-293 cells, a commonly used expression system to study plant ion channels [35–37], were employed as a more suitable alternative for this study. We subcloned the cDNA of MaAKT1 into pcDNA 6/His A eukaryotic vector and overexpressed it in HEK-293 cells. Whole-cell recording (Fig 2A) showed that, with K^+^ as the major cation in the solutions, overexpression of MaAKT1 generated a pronounced inwardly rectifying current (Fig 2B and 2C), with a reversal potential of approximately -10 mV, consistent with the equilibrium potential for K^+^. No significant inward rectifying currents were observed in control cells (Fig 2B and 2C).

To examine whether MaAKT1 channels share properties with Shaker K^+^ channel family members, we tested the response of the channel to Cs^+^ and Ba^2+^ and the permeability of the channel to monovalent cations. Whole-cell recording showed that MaAKT1 current could be blocked by 1 mM Cs^+^ (S4A Fig) and 10 mM Ba^2+^ (S4B Fig), reducing the current by 56.9 ± 5.8% and 89.1 ± 1.5%, respectively. Ionic replacement experiment showed that MaAKT1 is most permeable to K^+^, followed by NH_4_^+^ and Na^+^ (Fig 2D). To further characterize the single-channel properties of the channel, we carried out the single-channel recording using an inside-out patch configuration. A single-channel current with a conductance of 23.49 pS was recorded (Fig 2E and 2F). These properties are in accordance with AKT1 channels reported in other plant species, such as OsAKT1 [36].

### FSA inhibits MaAKT1-mediated inward rectifier K^+^ currents

**Fig 3:**
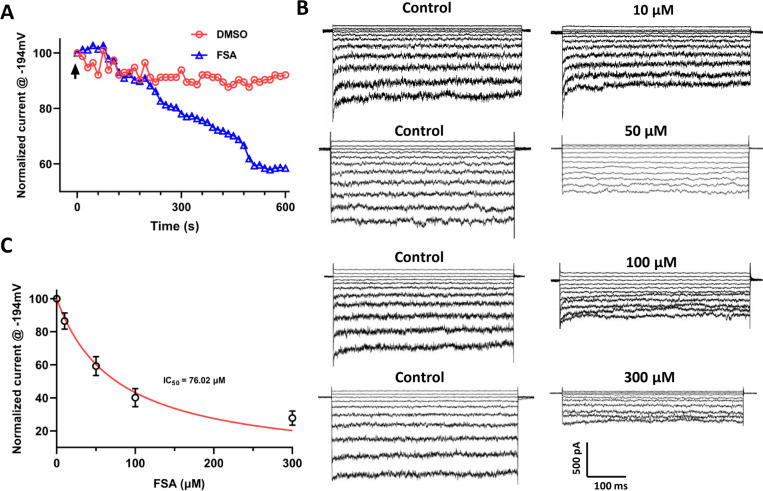
FSA inhibits MaAKT1 channels in a dose-dependent manner in HEK-293 cells. (**A**) Time course of normalized currents at −194 mV in FSA-treated and control groups. The arrow indicates the application time of FSA or DMSO (n = 4). (**B**) Representative whole-cell current traces showing the response of MaAKT1 currents to various concentrations of FSA. (**C**) Summarized currents at −194 mV, showing a dose-dependent inhibition of MaAKT1 current by FSA, with an IC_50_ of 76.02 μM (n = 5–10 for each data point).

To assess the effect of FSA on MaAKT1-mediated currents, we applied 50 μM FSA to the bath solution after recording the whole-cell current. Using a ramp voltage protocol from +6 mV to -194 mV at 15-s intervals, we observed that FSA application resulted in significant inhibition of MaAKT1 currents. The inhibition began at 2 minutes after FSA application and plateaued after 10 minutes; however, no significant current decay was detected in the DMSO-treated group (Fig 3A). We further explored the effects of different concentrations of FSA (10 μM, 50 μM, 100 μM, and 300 μM) on the current. The current inhibition rate at indicated concentrations demonstrated a pronounced dose-dependent inhibition, with an IC_50_ of 76.02 μM (Fig 3B and 3C). We then used FSA at a concentration near the IC_50_ (100 μM) in subsequent studies. At the maximum effect (measured mean current amplitude at −194 mV), the currents were inhibited by 71.20 ± 4.34% with 300 μM FSA.

### Oxidative stress involvement in FSA-induced inhibition of MaAKT1 channels

To explore the mechanism by which FSA inhibits MaAKT1-mediated K^+^ current, we first tested whether FSA interacts directly with MaAKT1 channels. FSA was washed out in the bath solution using a perfusion system, but no appreciable current recovery was observed (S5A and S5B Fig), suggesting that FSA does not inhibit the MaAKT1 channels through reversible binding. To further investigate whether FSA inhibited the channel depending on cytosolic signaling substance or not, we excised giant patches from cell membrane in an inside-out configuration to record macroscopic current. This configuration removes cytosolic signaling components, and FSA was applied directly to the intracellular membrane surface (S5C Fig). If FSA’s effect was mediated through intracellular signaling molecular, we would expect no inhibition in this configuration. However, FSA (100 μM) had no significant effect on the current, and only a marginal inhibition was observed at a higher concentration (500 μM) (S5D-S5F Fig), suggesting that FSA does not inhibit the MaAKT1 channel by direct interaction.

**Fig 4:**
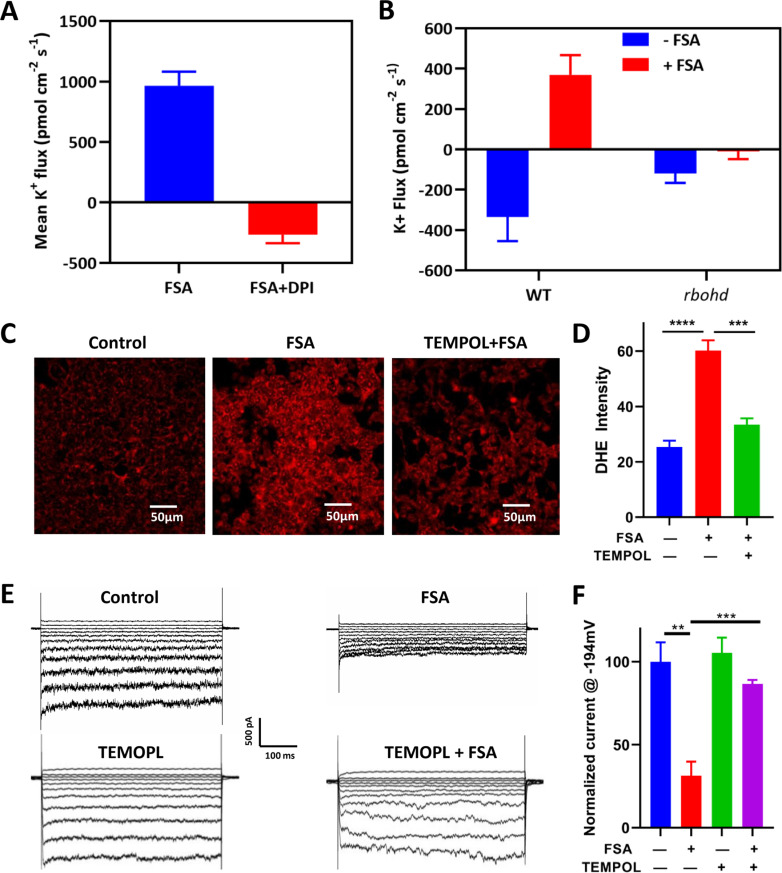
FSA inhibits MaAKT1 channels by increasing intracellular ROS levels. (**A**) Effect of 20 μM DPI (an RBOh inhibitor) on net K^+^ fluxes measured in epidermal roots cell of *Col-0* root. (**B**) Net K^+^ fluxes measured from epidermal root cells of *Col-0* and *rbohd* mutant root in response to 20 μM FSA. Data in (**A**) and (**B**) are presented as means ± SE (n = 10). (**C and D**) Representative DHE fluorescence images (**C**) and summarized DHE intensity (**D**) in control (left), FSA-treated (middle), and TEMPOL-pretreated, followed by FSA treatment (right) HEK-293 cells. Data are shown as means ± SE (n = 6). ***p<0.001 and ***p<0.0001 with one-way ANOVA followed by Newman-Keul’s test. (**E and F**) Representative whole-cell current traces (**E**) and summarized current density (**F**) of MaAKT1 current before and after FSA treatment in non-pretreated and TEMPOL-pretreated HEK-293 cells. Data are shown as means ± SE (n = 5). **p < 0.01 and ***p < 0.001 with one-way ANOVA followed by Newman-Keul’s test.

The butyl tail of FSA contributes its lipophilicity and facilitates its entry into the cellular interior. Like many other mycotoxins [38], FAS mediates its cytotoxicity through excessive reactive oxygen species (ROS) generation [39,40]. Our previous studies demonstrated that FSA increases intracellular ROS levels in banana ECSs [12]. In plants, ROSs are primarily generated by plasma membrane-bound NADPH oxidases, known as respiratory burst oxidase homolog (RBoh). To investigate whether ROS signaling plays a role in FSA-induced K^+^ efflux, we analyzed the effects of FSA in the presence or absence of Diphenylene iodonium (DPI), an RBoh inhibitor, on K^+^ fluxes in wild-type *Arabidopsis* Col-0 and *rbhod* mutant. FSA induced significant K^+^ efflux in Col-0, whereas DPI attenuated this effect (Fig 4A). Similarly, the *rbohd* mutant showed a reduced K^+^ efflux in response to FSA (Fig 4B). These results suggest that FSA inhibits MaAKT1 channels by elevating intracellular ROS levels.

Using DHE staining, we found that FSA treatment significantly increased ROS levels in HEK-293 cells, an effect largely prevented by TEMPOL, a ROS scavenger (Fig 4C). Electrophysiological measurements also support this hypothesis, as TEMPOL significantly attenuated the inhibitory effect of FSA on MaAKT1 channels (Fig 4C-4F). In addition, whole-cell recordings showed that two oxidants, H_2_O_2_, and diamide, produced a similar inhibitory effect on MaAKT1 channels, with concentration-dependent inhibition observed (S6 Fig). Maximum inhibition with 1 mM H_2_O_2_ and 100 μM diamide was 68.17 ± 6.45% (S6A and S6B Fig) and 72.19 ± 10.91% (S6C and S6D Fig), respectively, comparable to the inhibition observed with 300 μM FSA. Together, these findings suggest FSA inhibits MaAKT1 channels by elevating ROS levels.

### *S*-glutathionylation in the inhibition of MaAKT1 channels by oxidants

**Fig 5:**
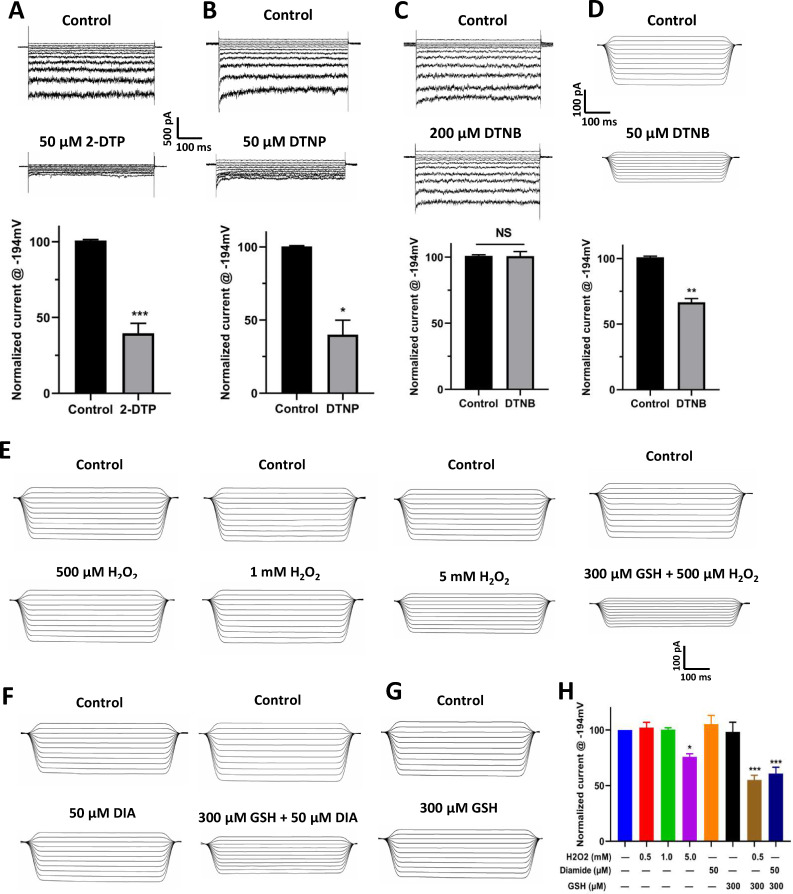
*S*-glutathionylation mediates the oxidants-induced inhibition of MaAKT1 channels. (**A-D**) The inhibition of MaAKT1 currents by thiol oxidants from the intracellular side. (**A-C**) Representative whole-cell current traces (top) and summarized current at −194 mV (bottom) showing MaAKT1 currents before and after application of the thiol oxidants, 2-DTP (**A**), DTBP (**B**), and DTNB (**C**). (**D**) Representative macroscopic current traces (top) and summarized current at −194 mV (bottom) showing changes in MaAKT1 currents following DTNB application. Data in (**A**) to (**D**) are shown as means ± SE (n = 5–6). Paired student’s t-test, ns denotes not significant, * p<0.05, **p < 0.01, and ***p < 0.001 compared with control. (**E-H**) GSH involvement in oxidant-induced inhibition of MaAKT1 channels. (**E-G**) Representative macroscopic current traces recorded in giant inside-out patches before and after application of H_2_O_2_ (**E**), diamide (**F**), with or without 300 μM GSH, and GSH alone (**G**). (**H**) Summary of the effects of H_2_O_2_, diamide, and GSH on MaAKT1 current. Data are shown as means ± SE (n = 4–7 for different treatments), *p < 0.05, ***p < 0.001 compared with control with one-way ANOVA followed by Newman-Keul’s test.

The inhibition of MaAKT1 by oxidants suggests the possible involvement of thiol oxidation, as diamide is an potent oxidizer of GSH [41], which forms intra- and intermolecular disulfide bonds in the presence of GSH [42]. To test this hypothesis, we applied several pyridine disulfides (PDSs), which selectively oxidize cysteine sulfhydryl group [43], to MaAKT1 channels. Whole-cell recordings showed that two cell membrane-permeable PDSs, 2,2-Dithiodipyridine (2-DTP) and 2,2’-dithiobis-5-nitropyridine (DTNP), inhibited the currents by 61.48 ± 6.66% and 60.01 ± 9.89%, respectively (Fig 5A and B). However, the membrane-impermeable PDS, 5,5-dithiobis-2-nitrobenzoic acid (DTNB, 200 μM) did not affect the current (Fig 5C, 100.72 ± 3.49%), even when applied at a concentration higher than those of 2-DTP and DTNP (50 μM). To investigate whether the differing effects of membrane-permeable and membrane-impermeable PDSs on MaAKT1 channels are attributable to their distinct potencies, we applied DTNB to the macroscopic currents in giant inside-out patches. In this configuration, low concentration of DTNB significantly inhibited MaAKT1 (Fig 5D, 35.28 ± 2.69%). To validate these findings in banana seedlings, we investigated the effects of 2-DTP and DTNP on K⁺ fluxes in the root tip zone. Using the Noninvasive Micro-test Technology (NMT) assay, we found that 2-DTP and DTNP treatments triggered significant K⁺ efflux in the root tip zone, similar to the effects of FSA (S7 Fig). These results suggest that the oxidant-induced inhibition of MaAKT1 may involve oxidation of cysteine thiol groups, with the crucial domain located on the cytosolic side of the channel.

The observed inhibitory effects of PDSs on MaAKT1 channels suggested that the inhibitory effect of oxidants may act through the introduction of a thiol adaptor to certain cysteine residue(s) rather than the direct formation of disulfide bonds. If this is the mechanism, cytosolic substances should be involved. As the action of PDSs targeting free cysteine sulfhydryl groups to form thiol moieties resembles *S*-glutathionylation, a well-established mechanism by which ROS regulates ion channels. We tested the role of *S*-glutathionylation in the regulation of MaAKT1 by oxidants using giant inside-out patches. In this configuration, H_2_O_2_ alone (500 μM and 1 mM) did not inhibit MaAKT1 significantly, but when applied with GSH, a lower concentration of H_2_O_2_ (500 μM) resulted in significant inhibition (45.9 ± 4.25%) (Fig 5E and 5H). Similarly, diamide alone did not affect the currents, while a significant inhibition (44.74 ± 4.75%) was observed when it was applied with GSH (Fig 5F and 5H). However, GSH alone did not affect the currents (Fig 5G and 5H). These results strongly suggest that *S*-glutathionylation plays a key role in the inhibition of MaAKT1 channels by oxidants.

**Fig 6:**
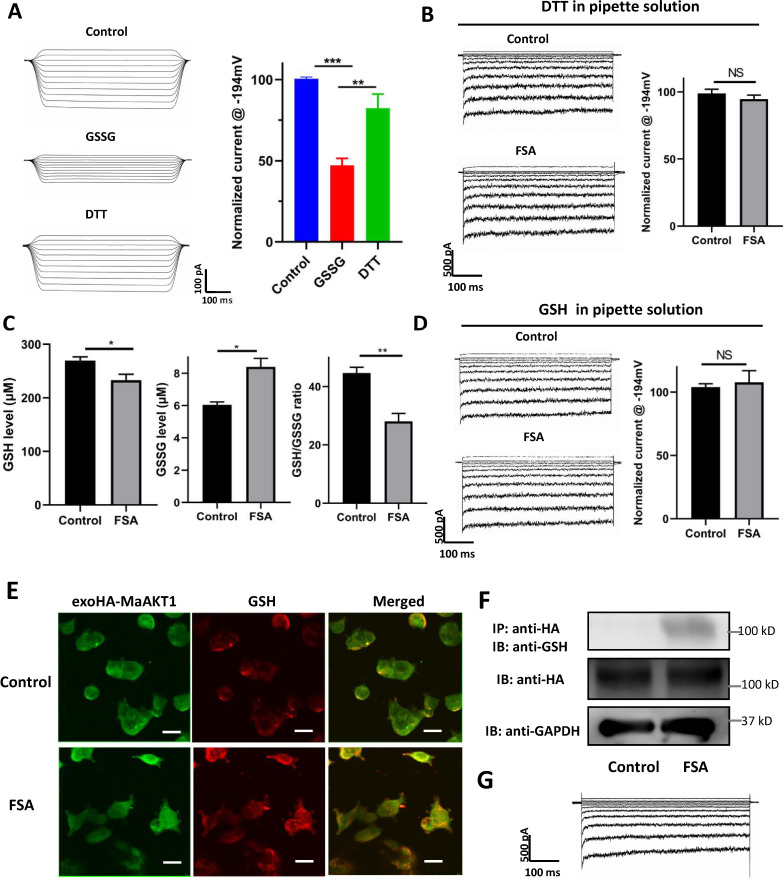
FSA inhibits MaAKT1 channels through *S*-glutathionylation. (**A**) DTT rescues MaAKT1 channels from inhibition by GSSG. (Left) Representative macroscopic current recorded in giant inside-out patches before (top), after 5 mM GSSG (middle), and after 5 mM GSSG followed by 5 mM DTT (bottom). (Right) Normalized current at −194 mV showing the effects of GSSG and DTT on MaAKT1 current. Data are shown as means ± SE (n = 6). One-way ANOVA followed by Newman-Keul’s test, **p < 0.01 and ***p < 0.001. (**B**) DTT in the patch pipette prevented MaAKT1 inhibition by FSA. Representative whole-cell current traces and summary of the preventive effect of DTT (5 mM) in the pipette on FSA-induced MaAKT1 inhibition (n = 3). Ns, not significant compared with control group. (**C**) FSA treatment decreases intracellular GSH level (left), increases GSSG level (middle), and reduces GSH/GSSG ratio (right) (n = 3). *p < 0.05 and **p < 0.01 compared with control. (**D**) GSH in the pipette prevents MaAKT1 inhibition by FSA. Representative whole-cell current traces and summary of the preventive effect of GSH (2 mM) in the pipette on FSA-induced MaAKT1 inhibition (n = 3). Ns, not significant compared with the control group. Data in (**B**) to (**D**) are shown as means ± SE and compared using Student’s t-test (**C**) or paired Student’s t-test (**B** and **D**). (**E**) Immunofluorescence showing that FSA treatment promotes the interaction between GSH (red) and MaAKT1 channels (green) (n = 5). Bar = 20 μm. (**F**) Co-IP results show that FSA treatment promotes the biochemical interaction between GSH and MaAKT1 channels (n = 3). (**G**) Representative whole-cell current recorded in HEK-293 cells overexpressing HA-tagged MaAKT1 channel.

To further validate this mechanism, we used the oxidized form of GSH (GSSG) as an *S*-glutathionylation inducer [42]. GSSG inhibited MaAKT1 currents in a dose-dependent manner, with 2 mM and 5 mM of GSSG inhibiting the currents by 34.61 ± 1.24% and 48.85 ± 3.47%, respectively (S8 Fig). Furthermore, the reducing agent dithiothreitol (DTT, 5 mM) restored the GSSG-mediated (5 mM) inhibition to 82.32 ± 8.73% of baseline (Fig 6A).

### Involvement of *S*-glutathionylation in the inhibitory effect of FSA on MaAKT1 channels

We assessed intracellular GSH and GSSG levels in HEK-293 cells and banana seedlings treated with FSA or vehicle. In HEK-293 cells, FSA treatment significantly reduced GSH levels from (269.65 ± 6.88) μM to (233.23 ± 10.78) μM and the GSH/GSSG ratio from 44.67 ± 1.98 to 28.09 ± 2.10, while concurrently increasing intracellular GSSG levels from (6.05 ± 0.18) μM to (8.40 ± 0.52) μM (Fig 6C). Similarly, in banana seedlings, FSA treatment reduced GSH levels from (6.20 ± 0.17) μM to (5.06 ± 0.20) μM and the GSH/GSSG ratio from 17.75 ± 1.17 to 10.18 ± 0.90, while increasing GSSG levels from (0.35 ± 0.02) μM to (0.47 ± 0.02) μM (S9 Fig). These results are consistent with the hypothesis that FSA induces oxidative stress.

To gain insight into whether FSA mediates its inhibitory effect through *S*-glutathionylation, we introduced GSH (2 mM) or DTT (5 mM) into the pipette solution during whole-cell recording, allowing the compounds to exchange with the cytosol for 5 minutes after pipette break-in the cells. The results indicated that FSA no longer inhibited MaAKT1 currents in the presence of GSH or DTT (Fig 6B and 6D). These electrophysiological observations indicate that FSA inhibits MaAKT1 via reversible *S*-glutathionylation of one or more sites within the channel, likely in or near the cytosol domain.

To further corroborate the role of *S*-glutathionylation in the inhibition of MaAKT1 by FSA, we conducted molecular biology experiments. An extracellularly HA-tagged MaAKT1 (exoHA-MaAKT1) construct was generated. Whole-cell recording revealed that the insertion of HA tag in the extracellular loop did not alter channel activity (Fig 6G). Immunofluorescence analysis of MaAKT1-overexpressing HEK-293 cells demonstrated that in control cells, GSH (red) did not interact with MaAKT1 channels (green), whereas FSA treatment facilitated a biochemical interaction (yellow) between GSH and MaAKT1 (Fig 6E). Co-immunoprecipitation experiments further supported this finding, showing that in cell lysate, HA-tagged MaAKT1 (~100 kDa) was clearly detected in both FSA-treated and untreated cells. After immunoprecipitation with an anti-HA antibody, GSH immunoreactivity co-localized with MaAKT1 in FSA-treated cells but was absent in DMSO-treated controls (Fig 6F). These results provide compelling evidence that FSA inhibits MaAKT1 channels through *S*-glutathionylation.

### Cysteine 202 residue is responsible for FSA-mediated *S*-glutathionylation of MaAKT1 channels

Given that MaAKT1’s structure has not been previously characterized, we used the online DeepTMHMM model to predict its transmembrane domains. The results revealed that MaAKT1 consists of six putative transmembrane segments (S10A Fig). Using these predictions, we constructed a secondary topology model (S10B Fig) and generated a closed-state structure of MaAKT1 based on the *Arabidopsis* AKT1 (AtAKT1) template using I-TASSER. The predicted structure indicates that MaAKT1 has a short intracellular N-terminal, six transmembrane segments, and a long intracellular C-terminal (S10C Fig).

**Fig 7:**
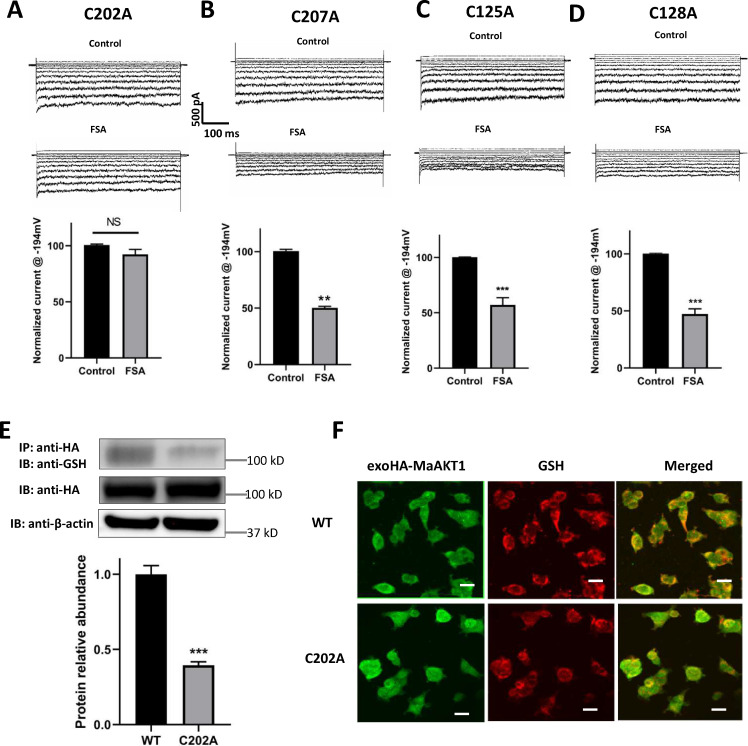
Cys202 is critical for FSA-induced *S*-glutathionylation of MaAKT1 Channels. (**A-D**) Representative whole-cell current traces and summaries showing the effect of FSA on different MaAKT1 mutants, including (**A**) C202A, (**B**) C207A, (**C**) C215A, and (**D**) C218A. Data are shown as mean ± SE (n = 3–7 for each mutant). Ns, not significant, **p < 0.01, ***p < 0.001 compared with control using paired Student’s t-test. (**E**) Co-IP results indicate that C202A mutation diminishes the biochemical interaction between GSH and MaAKT1 channels induced by FSA treatment. Data are shown as mean ± SE (n = 3). Student’s t-test, ***p < 0.001 compared with the WT group. (**F**) Immunofluorescence showing that C202A mutation reduces the biochemical interaction between GSH and MaAKT1 channels induced by FSA treatment (n = 5). Bar = 20 μm.

*S*-glutathionylation, a post-translational modification (PTM), involves the addition of a GSH moiety to cysteine residues. Therefore, identifying the cysteine(s) responsible for the *S*-glutathionylation of MaAKT1 by FSA is crucial, especially since MaAKT1 contains 13 cysteine residues. According to the structure model, cysteine residues are located in the C-terminal (1 residue), transmembrane segment 5 (4 residues), and the N-terminal (8 residues). Sequence alignment revealed that the cysteine residues in transmembrane segment 5 are highly conserved among homologs in different plant species (S2 and S10B Fig). Previous studies have shown that cysteines in the transmembrane domains play a key role in the *S*-glutathionylation of inward and outward rectifying K^+^ channels [44–46]. Therefore, we focused on the four cysteine residues in transmembrane segment 5: Cys202, Cys207, Cys215, and Cys218. We generated corresponding mutations (C202A, C207A, C215A, and C218A) and expressed them in HEK-293 cells. Whole-cell recordings revealed that none of the mutants altered channel function (Fig 7A-D). Consistent with prior observations that MaAKT1 current can be inhibited by thiol oxidants from the intracellular side, mutations in Cys215 and Cys218 did not affect the inhibition of the channel by FSA (Fig 7C and D, 42.8 ± 6.46% and 53.71 ± 4.51% inhibition, respectively). These residues are located near the cell surface, which likely prevents GSH incorporation. Unexpectedly, mutation in Cys207 also did not influence FSA’s inhibitory effect on the channel (Fig 7B, 49.73 ± 1.29% inhibition). However, when Cys202 was mutated to alanine, the inhibitory effect of FSA was substantially reduced (Fig 7A), with only 7.62 ± 4.4% inhibition observed. These findings suggested that Cys202 is the critical residue responsible for *S*-glutathionylation-mediated inhibition of MaAKT1 by FSA.

To further validate the role of Cys202 in FSA-induced *S*-glutathionylation of MaAKT1, we constructed an exoHA-MaAKT1-C202A mutant. Western blot analysis of whole-cell lysate from HEK-293 cells transfected with either wild-type (WT) or mutant MaAKT1 constructs revealed comparable expression level of MaAKT1 (Fig 7E), suggesting that the mutation did not alter the expression pattern of MaAKT1 protein. However, co-immunoprecipitation assays showed that the interaction between GSH and MaAKT1 was significantly reduced in the C202A mutant compared to WT MaAKT1 (Fig 7E). Immunofluorescence staining also demonstrated that the GSH-MaAKT1 interaction was substantially diminished in the C202A mutant (Fig 7F). Collectively, these data confirm that Cys202 is essential for the *S*-glutathionylation of MaAKT1 induced by FSA.

Given that MaAKT1 shares significant similarity with its homologues in other plant species, and that Cys202 is highly conserved (S2 and S10B Fig), we investigated whether the analogous cysteine residue in the *Arabidopsis* AKT1 channel (Cys191) also undergoes *S*-glutathionylation under oxidative stress. To explore this, AtAKT1 was exogenously overexpressed in HEK-293 cells and treated with FSA. The *S*-glutathionlyation of AtAKT1 was subsequently examined. Similar to MaAKT1, AtAKT1 also underwent *S*-glutathionylation in response to FSA treatment. Furthermore, mutation of Cys191 to alanine reduced the interaction between GSH and AtAKT1, as demonstrated by co-immunoprecipitation experiments and immunofluorescence staining (S11 Fig).

### Cys202 mutation alleviates *Foc* TR4 and FSA-induced yellowing symptoms

**Fig 8:**
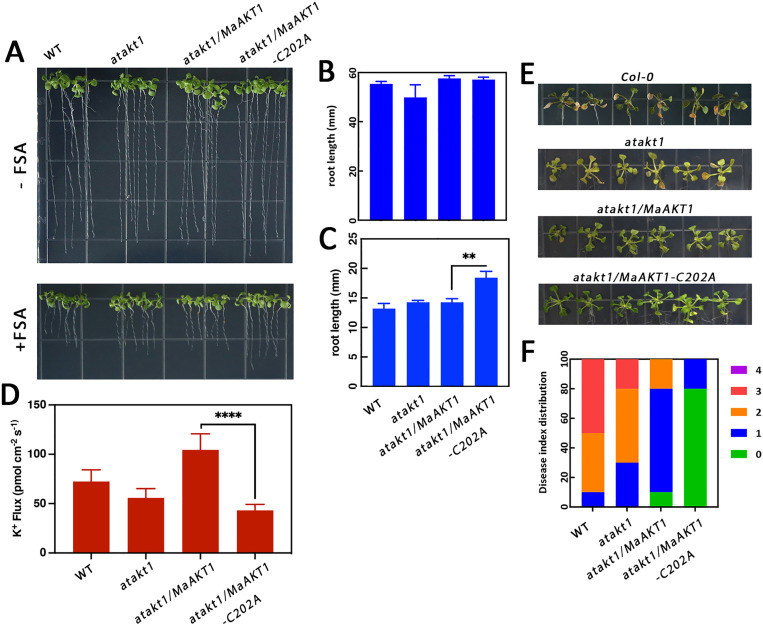
Cys202 mutation alleviates *Foc* TR4 and FSA-induced yellowing symptoms. (**A**) Representative phenotypes, (**B-C**) root length, and (**D**) K^+^ fluxes measured from epidermal root cells of WT, *atakt1* mutant, *atakt1/MaAKT1*, and *atakt1/MaAKT1-C202A* lines grown on MS medium with or without 10 μM FSA in *Arabidopsis*. (**E**) Disease symptoms and (**F**) DI distribution of WT, *atakt1* mutant, *akt1/MaAKT1*, and *akt1/MaAKT1-C202A* lines infected with Fo5176 at 7 dpi in *Arabidopsis*. Data in (**B**), (**C**), and (**D**) are shown as means ± SE (n = 10). Student’s t-test, **p < 0.01, ****p < 0.0001.

To examine the functional role of Cys202 in K^+^ efflux under FSA challenge, we genetically transformed MaAKT1 and MaAKT1-C202A constructs into *Arabidopsis atakt1* mutants using pBI121 vector. Under normal conditions (without FSA), neither MaAKT1 nor MaAKT1-C202A affected root growth. However, when exposed to FSA, root length in the *atakt1/MaAKT1-C202A* mutants was significantly greater than that in the WT, *atakt1*, and *atakt1/MaAKT1* mutants (Fig 8A–8C). Additionally, the *atakt1/MaAKT1-C202A* mutant exhibited significantly reduced K^+^ efflux in response to FSA compared to the *atakt1/MaAKT1* mutant (Fig 8D)

To determine whether Cys202 confers susceptibility to *F. oxysporum*, we inoculate *Arabidopsis* plants with a compatible *F. oxysporum* isolate (Fo5176). Seven dpi, both the *atakt1/MaAKT1* and *atakt1/MaAKT1-C202A* mutant plants exhibited fewer disease symptoms compared to the WT and *atakt1* plants, with the *atakt1/MaAKT1-C202A* group showing the highest resistance to fungi infection (Fig 8E and 8F). These results collectively suggest that FSA from *F. oxysporum* targets the Cys202 residue of the MaAKT1 channel to manipulate plant immunity and enhance its virulence.

## Discussion

AKT1 is a key K^+^ channel responsible for the uptake of K^+^ from soil into plant roots, playing a critical role in growth, development, and resistance to fungal attack. Malfunction of AKT1 channels impairs plant growth and reduces resistance to fungal infection [47]. In this study, we observed that banana plantlets infected with *Foc* TR4 or treated with FSA exhibited similar pathological symptoms to plants grown under the K^+^ deficiency conditions, including leaf yellowing, corm necrosis, and reduced growth of plantlets. Furthermore, FSA treatment led to reduced K^+^ content in roots. Patch-clamp studies revealed that FSA inhibited MaAKT1 activity, suggesting that FSA may induce net K^+^ loss as one of the mechanisms underlying the pathological progression in *Foc* TR4 infection.

*Foc* is a hemibiotrophic pathogen that employs various virulence factors to facilitate its infection of the host plants. FSA, a major virulent factor secreted by *Foc* TR4, induces the accumulation of ROS, which contributes to the development of disease symptoms in plants [9]. We previously reported that during *Foc* TR4 infection in banana plants, FSA acts as a pioneer molecule in pathogenesis, diffusing into host tissue before the arrival of the invading fungal hyphae [12]. In host cells, FSA disrupts the respiratory chain and induces mitochondrial ROS production, compromising the host’s immune response and increasing susceptibility to subsequent *Foc* TR4 infection [12]. However, the precise mechanisms by which FSA-induced respiratory dysfunction and ROS production enhance vulnerability to fungal attack remained unclear. Here, we found that FSA acts through ROS and *S*-glutathionylation to inhibit MaAKT1, leading to net K^+^ loss. Notably, transgenic plants carrying the Cys202 mutation, which prevents FSA inhibition of MaAKT1, exhibited reduced K^+^ loss from roots and less severe FSA-induced symptoms, including reduced root shortening and leaf yellowing. We therefore propose that FSA-induced inhibition of MaAKT1 contributes to the increased vulnerability of bananas to *Foc* TR4 infection, and that genetic modification of the MaAKT1 gene could represent a promising strategy to mitigate or overcome Fusarium wilt disease. Interestingly, similar mechanisms may also be involved the infection of rice by the fungal pathogen *Magnaporthe oryzae* [26]. *M. oryzae* can release an effector protein, AvrPiz-t, which suppress OsAKT1 and induces net K^+^ loss as a major mechanism to subvert plant immunity and enhance virulence [26].

Given the critical importance of AKT1 in plant growth and resistance to fungal attack, its activity must be tightly regulated. One well-documented mechanism involves calcineurin B-like proteins (CBL1/9) and CBL-interacting protein kinases (CIPK23), which regulates AKT1 in a Ca^2+^-dependent mechanism. In this pathway, CBL1/9 acts as a Ca^2+^ sensor, recruiting cytosolic CIPK23 to the plasma membrane, where it phosphorylates and activates the AKT1 channel [48,49]. Additionally, AKT1 can form heteromeric channels with the Shaker-like subunit AtKC1, which reduces K^+^ uptake as another regulatory mechanism [50,51]. In this study, we provided evidence for a novel AKT1 regulation pathway, namely, glutathionylation-mediated regulation. We demonstrate that FSA treatment elevates cellular ROS levels, promoting conversion of GSH to GSSG, which decreases the ratio of GSH/GSSG. GSSG, in turn, glutathionylates Cys202, inhibiting MaAKT1 activity. Several lines of evidence support the role of glutathionylation in regulating MaAKT1 activity: First, FSA treatment increased MaAKT1 glutathionylation as detected by western blot analysis. Second, subcellular staining showed increased colocalization of MaAKT1 and GSH signal following FSA treatment. Third, a point mutation of MaAKT1 changing Cys202 to an alanine abolished FSA-induced inhibition of MaAKT1 and reduced MaAKT1 glutathionylation, as confirmed by western blot and subcellular staining. More importantly, *Arabidopsis* plants carrying the Cys202 mutation exhibited longer root length, reduced K^+^ efflux in response to FSA, and displayed resistance to Fo5176 fungi attack and less severe yellowing symptom.

*S*-glutathionylation is a redox-mediated PTM in which a GSH moiety is added to the cysteine residues under oxidative stress [52]. It is known to regulate several ion channels in animals and humans, including ATP-sensitive K^+^ channels (Kir6.1) [44], inward-rectifier Kir4.1-Kir5.1 heteromeric channel complex [45], epithelial Na^+^ channel (ENaC) [53], and cystic fibrosis transmembrane conductance regulator (CFTR) Cl^-^ channel [42]. However, *S*-glutathionylation regulation has not been reported for plant ion channels. Our study represents the first example of *S*-glutathionylation regulation of a plant ion channel, and this type of regulation may not be restricted to the AKT1 channel in bananas but also occurs in its homologs in other plant species. It is noteworthy that two other types of thiol group modifications on cysteine residue, including *S*-nitrosylation and *S*-methioylation, have been reported to regulate plant outward rectifier K^+^ channels [46,54]. Therefore, our findings expand the understanding of redox-mediated regulation of cysteine residue in plant ion channels.

Efforts were made to identify the specific cysteine residue by which glutathionylation modification can exert an inhibitory effect on MaAKT1. Previous studies showed that in mammalian inward rectifier K^+^ channels Kir6.1/Kir5, *S*-glutathionylation modifies the cysteine residue near the transmembrane domain to regulate channel activity [44–46]. Therefore, we focused on Cys202, Cys207, Cys215, and Cys218, all of which are located in or near the transmembrane domain 5. Through a combination of site-directed mutagenesis, patch-clamp analysis, and immunoblotting analysis of glutathionylated MaAKT1, we identified an evolutionarily conserved site, Cys202, as the key cysteine involved in glutathionylation-mediated regulation of MaAKT1. This homologous site in AKT1 channels from other plant species also undergoes *S*-glutathionylation under oxidative stress. It has been well-accepted that the susceptibility of cysteine residues to redox modifications depends on two factors: the accessibility of the thiol within the protein’s 3D structure, and the reactivity of the cysteine, which is influenced by the neighboring amino acids [55]. Structural modeling using I-TASSER predicted that Cys202 is located near the intracellular side of the transmembrane domain 5, making it readily accessed to cytosolic GSH. In agreement with this, our patch-clamp analysis showed that *S*-glutathionylation occurs on the intracellular side of MaAKT1. Moreover, the presence of alanine and a phenylalanine residue near Cys202 enhances its propensity for *S*-glutathionylation, as suggested by bioinformatics studies [56]. Cys202 is also located near the S5 transmembrane domain, close to the ion-conducting pore, further supporting its role as the target of glutathionylation modification. Importantly, mutation of Cys202 completely abolished FSA-induced inhibition of MaAKT1, indicating that Cys202 is the critical site for glutathionylation-mediated regulation of MaAKT1. It is likely that glutathionylation of other cysteine residues, if present, does not contribute significantly to the inhibition of MaAKT1.

**Fig 9:**
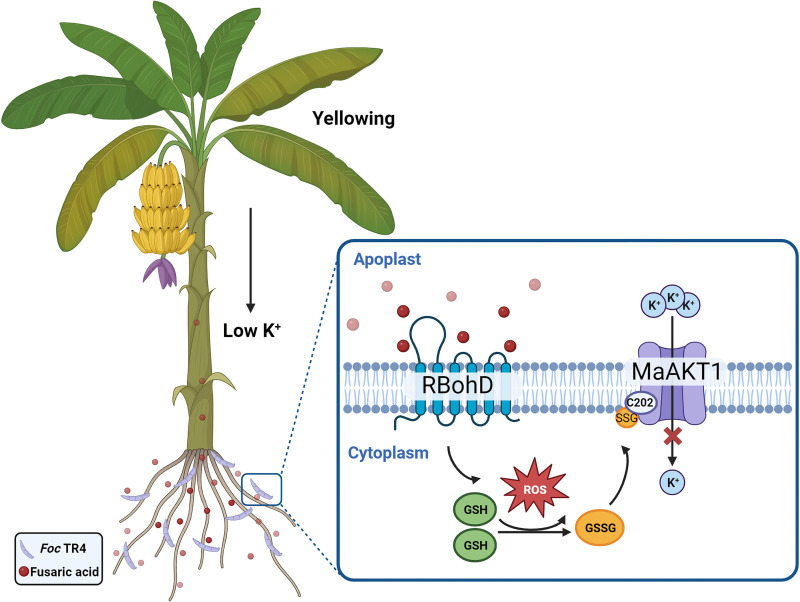
Working model of *Foc* TR4-induced suppression of MaAKT1-mediated K^+^ uptake. The secondary metabolite FSA secreted by *Foc* TR4 induces oxidative stress, leading to ROS accumulation in banana root cells through hijacking RBohD. This disrupts redox homeostasis, promoting the conversion of GSH to GSSG and inhibiting MaAKT1 channels via *S*-glutathionylation at Cys202 residue. Reduced MaAKT1 channel activity impairs K^+^ uptake and reduces K^+^ content in roots, ultimately causing yellowing symptoms.

In conclusion, this study reveals a novel mechanism by which the fungal toxin FSA inhibits MaAKT1 channels. We demonstrate that FSA, via ROS production, induces *S*-glutathionylation of Cys202, leading to inhibition of MaAKT1 channels, reduced K⁺ uptake, and net K⁺ loss, which may contribute to the exacerbation of *Foc* TR4-induced pathological damage in plants (see schematic in Fig 9). Our findings provide the first example of *S*-glutathionylation-mediated regulation of a plant ion channel and K⁺ nutrition, thereby enhancing the understanding of how FSA suppresses banana resistance to *Foc* TR4 infection.

## Materials and methods

### plant materials and treatments

The banana cultivar ‘Cavendish’ (AAA, *cv.* ‘Brazilian’) was grown under natural light conditions. *Arabidopsis* wild-type Col-0 (WT) was cultivated in a temperature-controlled glasshouse (16h light/8h dark cycle, 22°C, 40% humidity). The AKT1 T-DNA insertional mutants, *atakt1* (SALK_061400C) and *atrbohd* (SALK_120299), were obtained from ABRC. The MaAKT1 and MaAKT1-C202A cDNAs were cloned into the pBI121 vector and transformed into the akt1 mutant, generating the *atakt1/MaAKT1* and *atakt1/MaAKT1-C202A* lines. The transgenic plants were screened by hygromycin (Sigma) selection, and T3 generations were used for subsequent studies. For K^+^ stress treatment, Cavendish banana plantlets with 4-5 leaves (*c*. 22 cm in height) were grown in Murashige and Skoog (MS) medium without K^+^, while the control plants were supplied with standard MS solution. For FSA treatment, Cavendish banana plantlets were inoculated with 20 μM FSA. After 15 days, plantlets were photographed.

### Fungal strains and media

*Fusarium oxysporum* f. sp. *cubense* (*Foc*) ‘tropical’ race 4 (TR4) strain II5 (VCG01213) and *F. oxysporum* Fo5176 were used for inoculating ‘Cavendish’ banana and *Arabidopsis*, respectively. All *Foc* strains were cultivated on potato dextrose agar (PDA) at 28°C.

### Pathogenicity assay

Cavendish banana plantlets with 6-7 leaves (*c*. 25 cm in height) were inoculated with *Foc* TR4-II5 isolate. Conidia were harvested from 5-day-old cultures grown in potato dextrose broth (PDB). A spore suspension was prepared at a concentration of 10^5^ conidia/mL, with a total volume of 40 mL. This suspension was mixed with 4 kg of soil, achieving a final concentration of 1000 conidia/g of soil. Plantlets were then planted in inoculated soil mixture. For pathogenicity assessment, the disease index was assessed as our previous methods [12]: 0 (no symptoms), 1 (some brown spots in the inner rhizome), 2 (less than 1/4 of the inner rhizome showed browning), 3 (up to 3/4 of the inner rhizome showed browning), and 4 (entire inner rhizome and pseudostem were dark brown and dead). For infection on *Arabidopsis*, seedlings grown on 1/2 MS medium for 7 days were transferred to square dishes containing 1/2 MS medium. Five days later, each plant was inoculated with a piece of Fo5176 fungal plug (5 mM) [57]. Disease severity was classified as follows: 0 (no visible wilting or chlorosis), 1 (less than 1/4 of leaves wilted or chlorotic), 2 (1/4-1/2 of leaves wilted or chlorotic), 3 (1/2-3/4 of leaves wilted or chlorotic), and 4 (more than 3/4 of true leaves wilted or chlorotic) [58].

### Extraction of total RNA and RT-qPCR analysis

Total RNA from banana roots was extracted with a plant RNA Extraction Kit (AG, Changsha, China). First-strand cDNA was synthesized from the RNA using SuperScript II RNase H2 reverse transcriptase kit (Thermo Fisher Scientific). Quantitative PCR (qPCR) was performed using Power SYBR Green PCR Master Mix (Applied Accurate Biology). For each sample, three biological and three technical replications were performed. The banana MaTUB gene was used as an internal reference. Relative expression of MaAKT1 mRNA was calculated using 2^−ΔΔCt^ methods. Primer sequences are as follows:

MaAKT1: F 5’GGGTTCGATGCGCAAAACTT3’; R 5’CCGTCGTAAGGGTGGTGATT3’,
MaTUB: F 5’TGTTGCATCCTGGTACTGCT3’; R 5’GGCTTTCTTGCACTGGTACAC3’.

### Analysis of K^+^ content and Net K^+^ flux

K^+^ content was measured as described previously [48]. Briefly, Cavendish banana plantlets were dried and grounded into powder. All samples were ashed in a muffle furnace at 300°C for 2 hours and 575°C for 12 hours, then dissolved in 0.1 M HCl. K^+^ content was determined using an atomic emission spectrometer (Agilent 4100-MP AES, Santa Clara, CA, USA). Net K^+^ flux was measured in the elongation zone (*c*. 800 μm from the tip of *Arabidopsis* roots and *c.* 1000 μm from the tip of banana roots) using a noninvasive microsensing system (NMT Physiolyzer; Xuyue Science and Technology LLC). Briefly, *Arabidopsis* seedlings were grown in a Petri dish for 7 days. For pretreatments, plants were pretreated with measuring solution (2.5 mM KNO_3_, pH 6.0) or plus 0.02 mM Diphenylene iodonium (DPI, an NADPH oxidases inhibitor) for 1 hour. After pretreatment, FSA was added to the measuring solution to a final concentration of 20 μM. Data was recorded using iMFluxes V2.0 software (Younger USA LLC, Amherst, MA 01002, USA).

### Phylogeny analysis

The amino acid sequences of all AKT1 proteins were downloaded from Uniprot database (https://www.uniprot.org/). Sequences alignment was performed using ClustalX 2.1 software (Informer Technologies, Inc), and the alignment of MaAKT1 with AKT1 channels from other plant species was visualized using ESPrint 3.0. Phylogenetic analysis was conducted using the maximum likelihood (ML) method by Mega 7.0, with 1000 bootstrap tests.

### Constructs and mutagenesis

The complementary DNAs (cDNAs) of the MaAKT1 and AtAKT1 channels were synthesized, sequenced, and subcloned into pcDNA 6/His A and pcDNA3.1 eukaryotic vectors, respectively. For molecular biology studies, extracellular hemagglutinin (HA)-tagged MaAKT1 (exoHA-MaAKT1) and Flag-tagged AtAKT1 (exoFlag-AtAKT1) constructs were generated as previously described for inserting HA tag into extracellular loop of human TRPC3 ion channel [59]. Briefly, the TACCCCTACGACGTGCCCGACTACGCC sequence encoding YPYDVPDYA and GACTACAAAGACGATGACGACAAG encoding DYKDDDDK were inserted into MaAKT1 between Pro162 and Pro163 and AtAKT1 between Ser153 and Ser154, respectively, resulting in the addition of HA or Flag epitope in the putative second extracellular loop. For immunofluorescence studies, exoHA-MaAKT1 was subcloned into pcDNA6-Myc-HisB vector (Suchow SynbioTech, Suzhou, China) using Kpnl and Notl restriction enzymes. Mutagenesis was performed with site-directed mutagenesis kits (Stratagene) according to the manufacturer’s instructions. All constructs and mutations were verified by DNA sequencing. The functional impact of the HA-tag insertion and mutation on MaAKT1 channel activity was evaluated prior to further experiment.

### Cells and transfections

Human embryonic kidney (HKE-293) cells (ATCC) were used as a heterogeneous expression system for functional and molecular biology studies of MaAKT1. The cells were cultured in Dulbecco’s modified Eagle medium (DMEM, GIBCO) with 4.5g/L D-glucose, supplemented with 10% fetal bovine serum (GIBCO) and 1% antibiotic-antimycotic (GIBCO) in the humidified environment at 37°C with 5% CO_2_. The cells were passaged every 3 days and used at passage number less than 50. For transfection, cells were plated in 35-mm Petri dish to 60% confluence and transfected with 2 μg of MaAKT1 or AtAKT1 cDNA with 5 μg of Lipofectamine 2000 (Invitrogen Inc., Carlsbad, CA) according to manufacturer’s instructions. Twenty-four hours post-transfection, cells were transferred to 12-mm coverslips for patch-clamping and immunofluorescence staining, and experiments were conducted within 48 hours. For co-immunoprecipitation studies, two 10-cm dishes of HEK-293 cells were transfected with 8 μg of cDNA and 24 μg of Lipofecatmine 2000 (Invitrogen Inc., Carlsbad, CA). Forty-eight hours post-transfection, cells were treated with 100 μM FSA for 30 minutes and then harvested.

### Electrophysiological studies

Successfully transfected cells were identified by green fluorescence under a fluorescence microscopy. The pharmacological and electrophysiological properties of MaAKT1 channels were assessed using patch-clamp techniques at room temperature (21–23°C) in various recording configurations. For whole-cell recordings, pipettes with a resistance of 2–4 MΩ were used. The compositions of the pipette and bath solution were prepared as previously described [60]. The pipette solution contained (in mM): 150 K-gluconate, 2 MgCl_2_, 10 EGTA, 10 Hepes/Tris pH 7.2, π = 300 mosmol/kg, adjusted with D-mannitol. The bath solution contained (in mM) 100 K-gluconate, 10 Hepes/Tris pH 7.4, π = 300 mosmol/kg, adjusted with D-mannitol. Step pulses were applied from +6 mV to −194 mV in 20 mV decrements for 500 ms, with a holding potential of -34 mV. For single-channel recordings, pipettes with a resistance of 12–15 MΩ were used to record current from small inside-out patches excised from the cell membrane. Giant inside-out patches were excised using pipettes with a resistance of 4–6 MΩ to investigate whether FSA inhibits MaAKT1 via intracellular mechanisms. In this mechanism, intracellular signaling molecular is lost and FSA is applied to intracellular membrane surface [61]. For this recording, the bath and pipette solutions were equivalent to the pipette and external solutions used in whole-cell recordings, respectively [61]. Ionic permeability experiments were performed by replacing K^+^ in the bath solution with equal molar concentrations of NH₄⁺ or Na⁺. Recordings were acquired with an EPC10 amplifier (HEKA Elektronik, Lambrecht, Germany), and data were low-pass filtered at 3 kHz with an 8-pole Bessel filter and digitized at 10 kHz. Capacitance and access resistance were continuously monitored and compensated to minimize voltage errors. Single-channel currents were recorded at a constant voltage for a 60-second period. Prior to analysis, traces from macroscopic and single-channel recordings were filtered further at 30 and 100Hz, respectively. Data analysis and curve fitting was performed using Origin Pro 8.5 (OriginLab Corporation, Northampton, MA). The slope conductance was determined by linear regression with Sigmaplot 10.0 (Jandel Scientific, San Diego, CA). The current-voltage (i–V) relationship was constructed by plotting current density (pA/pF) against voltage. The inhibition percentage of MaAKT1 currents by chemical treatment at -194 mV was calculated as follows: Inhibition% = (I_0_ − I_10 min_)/I_0_, where I_0_ is the currents before chemical application, and I_10 min_ is the currents at 10 minutes after chemical application.

### Immunocytochemistry

Immunocytochemistry was performed on HEK-293 cells transfected with exoHA-MaAKT1 and exoHA-MaAKT1(C202A) plasmids subcloned into pcDNA6-Myc-HisB, or exoFlag-AtAKT1 and exoFlag-AtAKT(C191A) plasmids subcloned into pcDNA3.1 vector as described previously [62]. Twenty-four hours after transfection, cells were transferred to poly-L-lysine coated coverslips (Thermo Fisher Scientific). After 12 hours, cells were treated with 100 μM FSA or dimethyl sulfoxide (DMSO, 0.1%) for 30 minutes. The supernatant was discarded, and cells were washed three times with PBS, followed by fixation with 4% paraformaldehdyde (PFA). Permeabilization was carried out with 0.25% Triton X-100, and non-specific binding was blocked with 1% BSA for 30 minutes prior to incubation with primary antibodies. For double-staining, cells were incubated with antibodies against HA tag (51064-2-AP, Proteintech Group, 1:100) and glutathione (GSH, MA1-7620, Thermo Fisher Scientific, 1:100) or anti-Flag (AF0036, Beyotime Biotechnology, 1:100) and anti-GSH for 2 hours at room temperature (21–23°C), followed by incubation with corresponding secondary fluorescence antibodies (A32731 and A32773, Thermo Fisher Scientific, 1:100) for 1 hour at room temperature. After washing with PBS for 3 times, coverslips were mounted on glass slides with anti-fade mounting oil (Beyotime Biotechnology, Shanghai, China) and visualized under an FV-1200 Olympus confocal microscopy.

### Co-immunoprecipitation

Co-immunoprecipitation was performed to identify the biochemical interaction between GSH and MaAKT1, or interaction between GSH and AtAKT1following FSA treatment, as previously described [55]. Two 10-cm dishes of HEK-293 cells were transfected with exoHA-MaAKT1, exoHA-MaAKT1(C202A), exoFlag-AtAKT1, or exoFlag-AtAKT(C191A) plasmids. Forty-eight hours post-transfection, cells were treated with 100 μM FSA or vehicle. Cells were then washed with chilled PBS and lysed with IP lysis buffer (20 mM Tris, pH 7.5, 150 mM NaCl, 1% Triton X-100, 50 mM N-ethylmaleimide 1% phenylmethylsulfonyl fluoride) (Beyotime Biotechnology, Shanghai, China). Protein concentration was determined with DC Protein Assay Reagent (Bio-Rad, Richmond, CA, USA) according to the manufacturer’s instructions. Forty microliters of each protein sample were preserved as input, while 2.5 mg of protein was incubated with 60 μL of protein-G agarose slurry and 2.5 μg of anti-rabbit IgG at 4°C for 2 hours to reduce non-specific binding. The supernatant was transferred to new tubes and incubated with 5 μg of anti-HA tag antibody or anti-Flag antibody for 12 hours at 4°C under gentle rotation. Following incubation with protein-G agarose for 4 hours, the beads were collected and washed with cold PBS 4 times. The beads were then resuspended in 30 μL of 1× non-reducing loading buffer and heated at 100°C for 3 mins. Proteins were separated by 10% SDS-PAGE, transferred to PVDF membranes, and blocked with quick-blocking buffer (Beyotime Biotechnology, Shanghai, China) at room temperature for 30 minutes. The membrane was incubated overnight at 4°C with the primary antibodies against the HA tag (51064-2-AP, Proteintech Group, 1:1000), Flag tag (F1804, Sigma, 1:2000), GSH (MA1-7620, Thermo Fisher Scientific, 1:1000), β‐actin (66009-1-Ig, Proteintech Group, 1:1000) or GAPDH (Affinity AF7021, Affinity Biosciences, 1:2000), After washing with TBS‐T, the membrane was incubated with horseradish peroxidase-conjugated secondary antibody (NA931V and NA934V, GE Healthcare, 1:5000) for 1 hour at room temperature. Blots were detected using an ECL kit (GE Healthcare) and visualized with the ChemiDoc XRS Plus system (Bio‐Rad, Richmond, CA, USA). Band intensities were quantified by Image J software (National Institutes of Health, Bethesda, MD).

### Intracellular ROS detection

HEK-293 cells treated with vehicle, FSA, or TEMOPL + FSA were stained with the cell-permeable fluorescent dye dihydroethidium (DHE) (Beyotime Biotechnology, Shanghai, China), a ROS indicator. Briefly, cells were loaded with 5 μM DHE for 30 mins, followed by PBS washing to remove excessive dye. DHE fluorescent were visualized using confocal microscope at an excitation wavelength of 594 nm. The intensity of fluorescence was quantified with Image J software (National Institutes of Health, Bethesda, MD).

### Intracellular GSH and GSSG detection

Intracellular GSH and oxidized form of GSH (GSSG) levels were measured using a commercially available kit (Beyotime Technology, Shanghai, China) according to the manufacturer’s instructions. Briefly, HEK-293 cells treated with FSA or vehicle were washed with chilled PBS and harvested. Banana seedlings were treated with 20 μM FSA for 7 days, and the root tips were harvested and processed. Samples were mixed with 5% metaphosphoric acid at the ratio of 1:3 (v/v), which were then subjected to two frozen-thawed cycles with liquid nitrogen and at 37°C. After centrifugation, the supernatant was collected for GSH and GSSG detection. The levels of total and GSSG were determined by the standard curve of TNB at 412 nm, with a multimode microplate reader (Bio-Rad, Richmond, CA, USA).

### Structural modeling

The transmembrane domain of MaAKT1 was predicted using the online DeepTMHMM Model (Technical University of Denmark). The native MaAKT1 structure in its closed state was modeled by I-TASSER protein structure prediction [63], using *Arabidopsis* AKT1(AtAKT1) channel as a template. Structural images were generated with the PyMol molecular graphics system.

### Data analysis

Data were presented as Mean ± S.E. Statistical comparisons were conducted using paired or unpaired Student’s t-tests or one-way ANOVA followed by Newman-Keul’s test, and statistical significance was accepted when p < 0.05.

## Supporting information

S1 Fig Transcriptional changes of MaAKT1 in response to *Foc* and FSA treatment. (**A**) Expression levels of MaAKT1 in banana roots infected with *Foc* race1 and TR4. (**B**) Transcript levels of MaAKT1 in FSA-treated banana plantlets at indicated time points (n = 3).
(TIF)

S2 Fig Multiple sequence alignment of MaAKT1 and its homologs from other plant species. Sequences were aligned using MEGA 7.0. The alignment included 8 AKT1 proteins: MaAKT1 from banana (*Musa acuminata*, XP_009386140.1), AtAKT1 from *Arabidopsis* (*Arabidopsis thaliana*, At2g26650), ZmAKT1 from maize (*Zea mays*, ZEAMMB73_Zm00001d011473), OsAKT1 from rice (*Oryza sativa*, Os01g0648000), TaAKT1 from wheat (*Triticum aestivum*, CFC21_041608), StAKT1 from potato (*Solanum tuberosum*, NP_001275347.1), GmAKT1 from soybean (*Glycine max*, GLYMA_05G010600) and SlAKT1 from tomato (*Solanum lycopersicum*, NP_001234258.2).
(PDF)

S3 Fig Maximum likelihood tree based on MaAKT1 and its homologs from other plant species. (TIF)

S4 Fig Sensitivity of MaAKT1 channels to K^+^ channel blockers. Representative MaAKT1 current traces and summary showing current changes before (top) and after (bottom) Cs^+^ (n = 5) and Ba^2+^ (n = 5) application, Data are shown as mean ± SE. Paired Student’s t-test, ****p<0.0001 compared with control.
(TIF)

S5 Fig FSA does not inhibit MaAKT1 channels through direct interaction. (**A** and **B**) Representative whole-cell current traces and summary showing the effect of FSA and washout on MaAKT1 currents. Data are shown as mean ± SE (n = 4), **p < 0.01 compared with control. Data are shown as mean ± SE (n = 4), **p < 0.01 compared with control (one-way ANOVA followed by Newman-Keul’s test). (**C)** Diagram illustrating the inside-out recording in giant patches excised from the cell membrane of HEK-293 cells overexpressing MaAKT1 channels, with FSA applied directly to the intracellular membrane surface via a pipette. **(D)** Representative macroscopic MaAKT1 current traces in giant patches excised from MaAKT1-overexpressed HEK-293 cells before and after 100 μM and 500 μM FSA. (**E**) Normalized current at -194 mV after treatment with 100 μM and 500 μM FSA in giant patches. Data are shown as mean ± SE (n = 5 for each concentration), *p < 0.05 compared with control (one-way ANOVA followed by Newman-Keul’s test).
(TIF)

S6 Fig Inhibition of MaAKT1 currents by H_2_O_2_ and diamide in HEK-293 cells. **(A)** Representative whole-cell current traces recorded before (left) and after (right) application of different concentrations of H_2_O_2._ (**B**) Normalized currents at −194 mV showing dose-dependent inhibition of MaAKT1 current by H_2_O_2_ with an IC_50_ of 234.7 μM (n = 3–7). (**C**) Representative current traces recorded before (left) and after (right) application of different concentrations of diamide. (**D**) Normalized currents at −194 mV showing dose-dependent inhibition of MaAKT1 current by diamide, with an IC_50_ of 29.22 μM. Data are shown as mean ± SE (n = 4–7).
(TIF)

S7 Fig Effects of FSA and PDSs on net K⁺ fluxes in the root tip zone of banana seedlings. Net K⁺ fluxes were measured from banana root tips treated with FSA (20 μM), 2-DTP (50 μM), or DTNP (50 μM) using non-invasive micro-test technology (NMT). Data are shown as means ± SE (n = 6). Negative values indicate K⁺ efflux from root cells.
(TIF)

S8 Fig GSSG inhibits MaAKT1 channels in a dose-dependent manner. (**A and B**) Representative macroscopic current traces recorded in giant inside-out patches before and after the application of 2 mM (**A**, n = 5) and 5 mM GSSG (**B**, n = 9). (**C**) Normalized current at -194 mV showing the effect of different doses of GSSG on MaAKT1 current. Data are shown as mean ± SE, *p < 0.05, ***p < 0.001, and ****p < 0.0001(one-way ANOVA followed by Newman-Keul’s test).
(TIF)

S9 Fig Intracellular levels of GSH and GSSG in banana seedlings treated with FSA. (A) GSH levels, (B) GSSG levels, and (C) GSH/GSSG ratio in the roots of banana seedlings treated with 20 μM FSA for 7 days. Data are presented as means ± SE (n = 6). **p < 0.01, Student’s t-test.
(TIF)

S10 Fig Transmembrane topology prediction and structural modeling for MaAKT1 channel. (**A**) Predicted transmembrane domains of MaAKT1 channel using the DeepTMHMM Model. (**B**) Secondary structure of the MaAKT1 channel based on predicted transmembrane domains. (**C**) Predicted closed-state structure of MaAKT1 by the I-TASSER protein structure prediction server using AtAKT1channel as a template.
(TIF)

S11 Fig FSA induces *S*-glutathionylation in AtAKT1 channels. Co-IP results showing that FSA treatment promotes the biochemical interaction between GSH and AtAKT1 channels, and C191A mutation diminishes the interaction (n = 4). **p < 0.01, one-way ANOVA followed by Newman-Keul’s test. **(B)** Immunofluorescence images showing that FSA treatment promotes the interaction between GSH (red) and AtAKT1 channels (green) (n = 5), and C191A mutation diminishes the interaction. Bar = 20 μm.
(TIF)
