# Correction: Fusaric acid-mediated *S*-glutathionylation of MaAKT1 channel confers the virulence of *Foc* TR4 to banana

**DOI:** 10.1371/journal.ppat.1014013

**Published:** 2026-03-04

**Authors:** The PLOS Pathogens Staff

There are errors in the author affiliations. The correct affiliations are as follows:

Jun Zhang^1,2^, Siwen Liu^3,4,5^, Wenlong Yang^3^, Yanling Xie^3^, Chuange Shao^3^, Zhi-Ren Zhang^1^, Chunyu Li^3,4,5^, Xiaoqiang Yao^2,6^

1 Department of Cardiology, The First Affiliated Hospital of Harbin Medical University, NHC Key Laboratory of Cell Transplantation, Key Laboratories of Education Ministry for Myocardial Ischemia Mechanism and Treatment, Harbin, China, 2 School of Biomedical Sciences, Li Ka Shing Institute of Health Science, The Chinese University of Hong Kong, Hong Kong, China, 3 Institute of Fruit Tree Research, Guangdong Academy of Agricultural Sciences, Key Laboratory of South Subtropical Fruit Biology and Genetic Research Utilization, Ministry of Agriculture and Rural Affairs, Guangdong Provincial Key Laboratory of Tropical and Subtropical Fruit Tree Research, Guangzhou, China, 4 Guangdong Laboratory for Lingnan Modern Agriculture, Guangzhou, China, 5 Maoming Branch, Guangdong Laboratory for Lingnan Modern Agriculture, Maoming, China, 6 Centre for Cell & Developmental Biology, School of Life Sciences, The Chinese University of Hong Kong, Hong Kong, China.

The publisher apologizes for the error.
