# Supplementary figures and images for: Fusaric acid-mediated S-glutathionylation of MaAKT1 channel confers the virulence of Foc TR4 to banana
Source: PLoS Pathog. 2025 Apr 9;21(4):e1013066. doi: 10.1371/journal.ppat.1013066 (PMC12040275; doi:10.1371/journal.ppat.1013066)

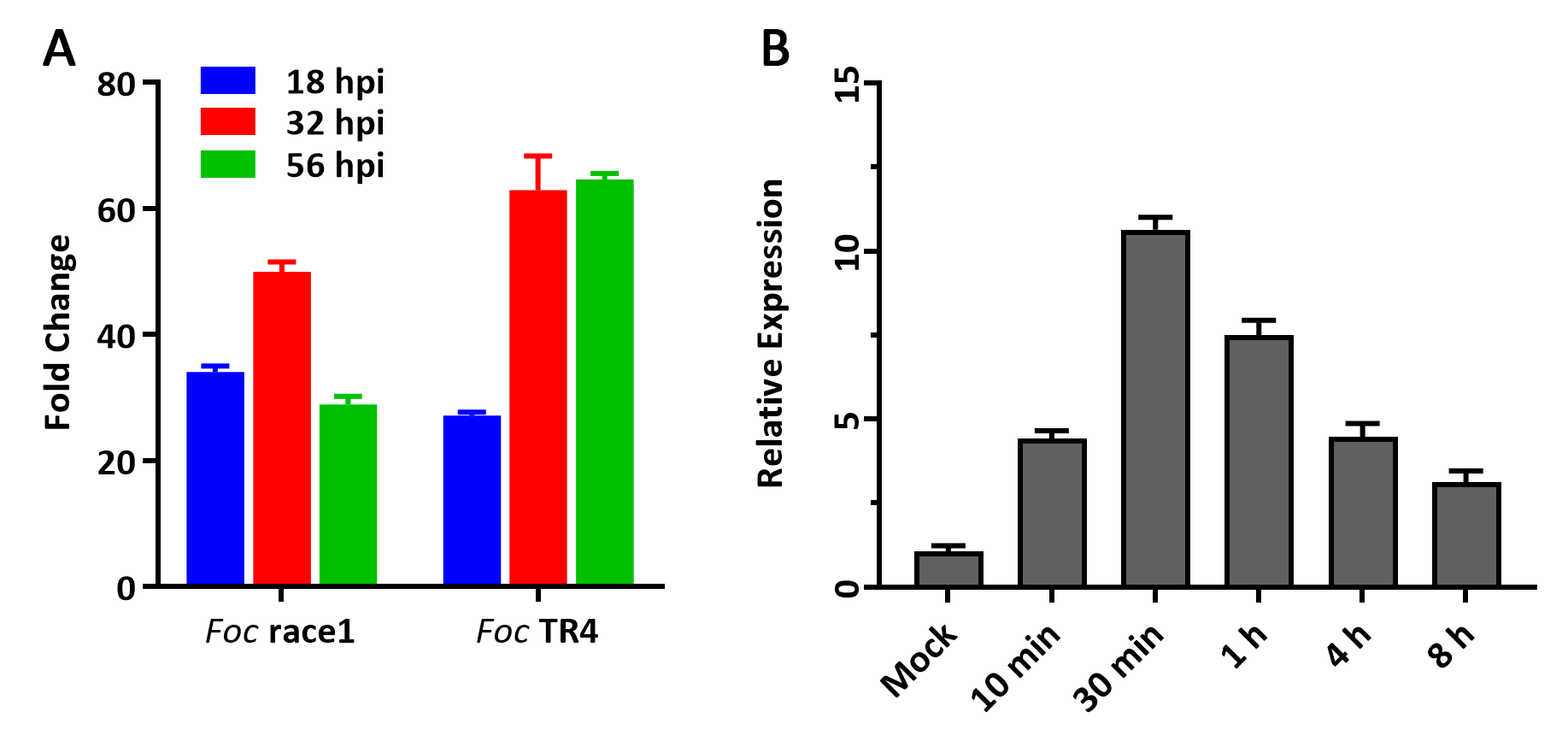

Supplement: S1 Fig — (A) Expression levels of MaAKT1 in banana roots infected with Foc race1 and TR4. (B) Transcript levels of MaAKT1 in FSA-treated banana plantlets at indicated time points (n = 3). (TIF) [file ppat.1013066.s001.tif]

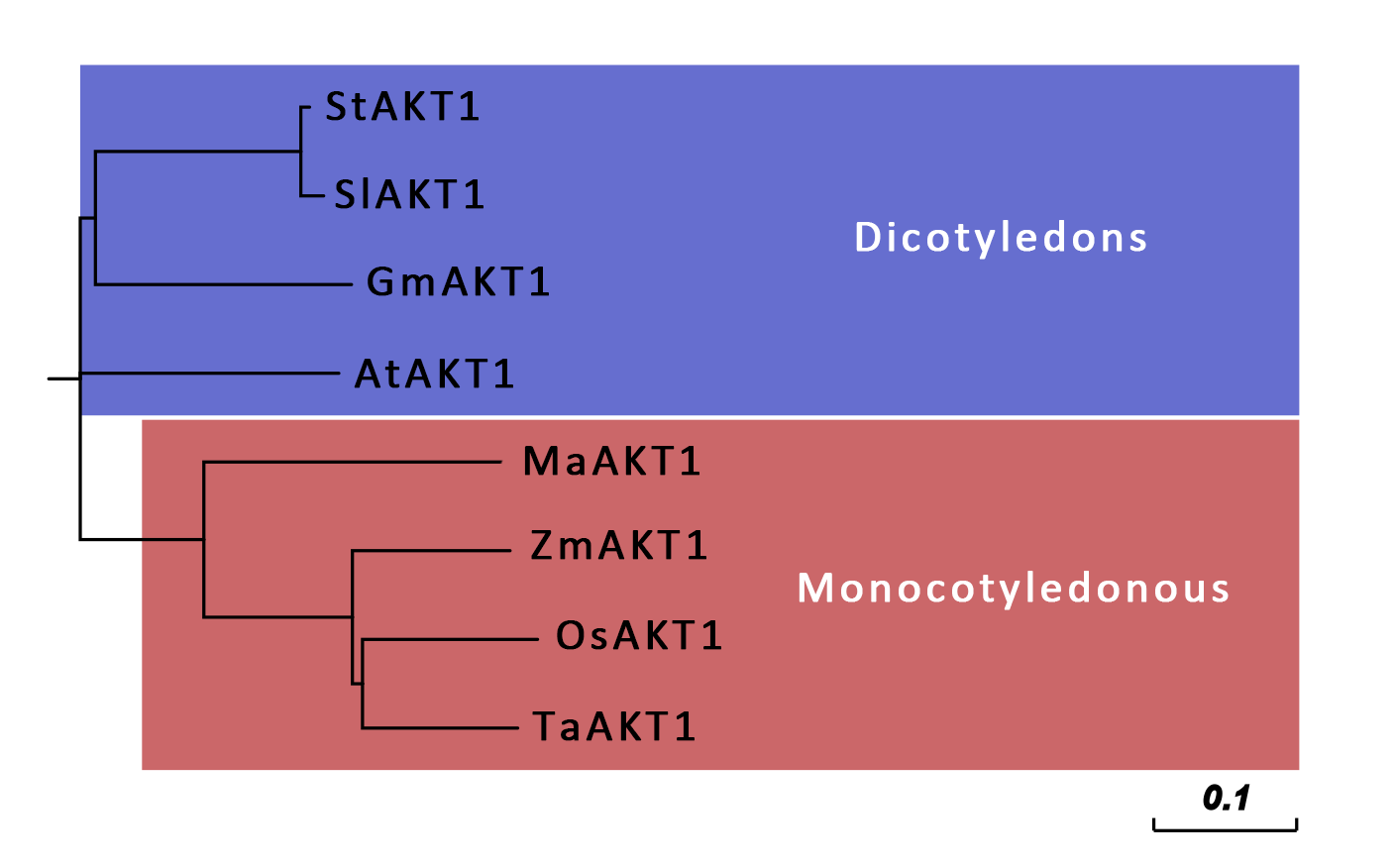

Supplement: S3 Fig — (TIF) [file ppat.1013066.s003.tif]

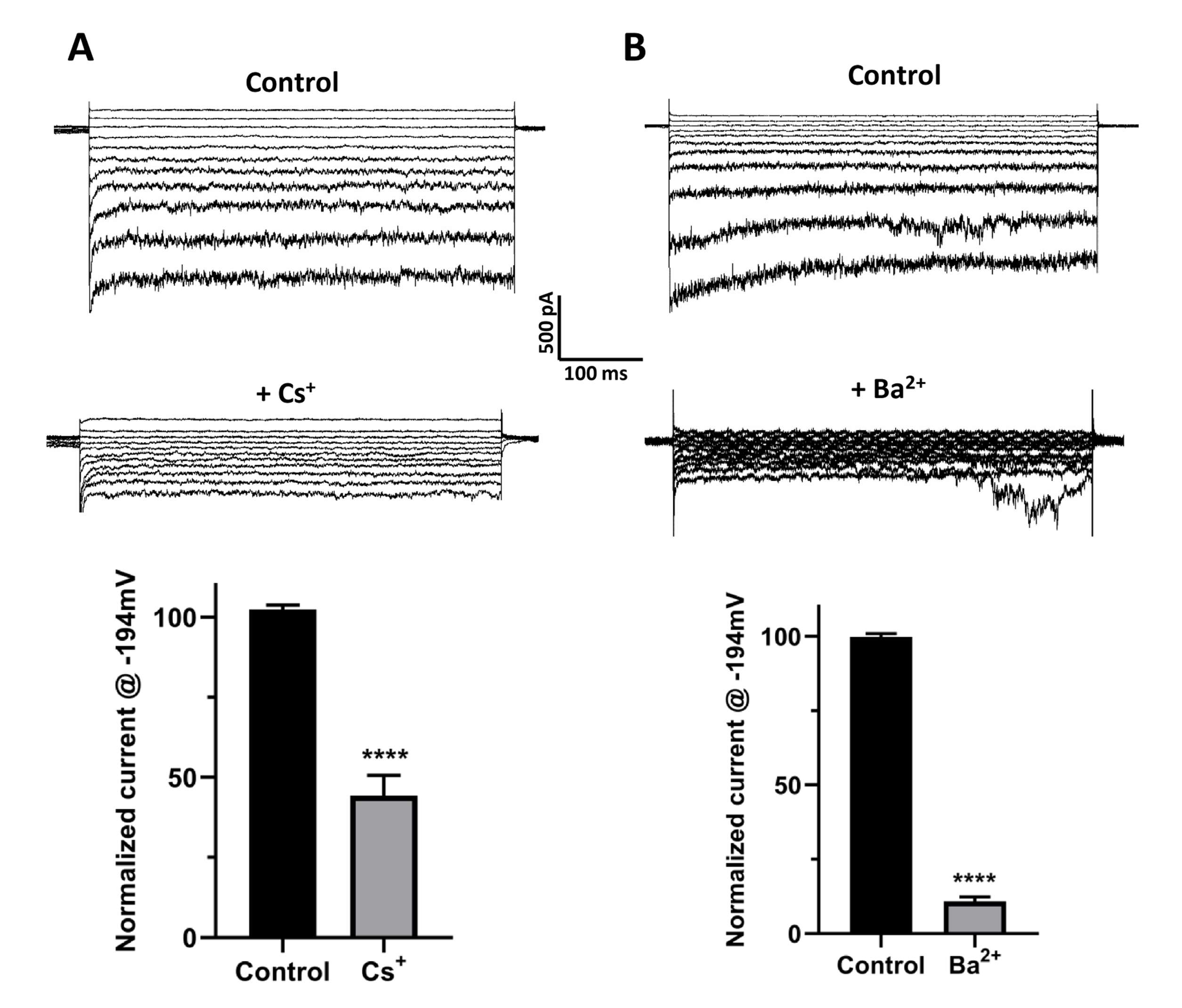

Supplement: S4 Fig — Representative MaAKT1 current traces and summary showing current changes before (top) and after (bottom) Cs+ (n = 5) and Ba2+ (n = 5) application, Data are shown as mean ± SE. Paired Student’s t-test, ****p<0.0001 compared with control. (TIF) [file ppat.1013066.s004.tif]

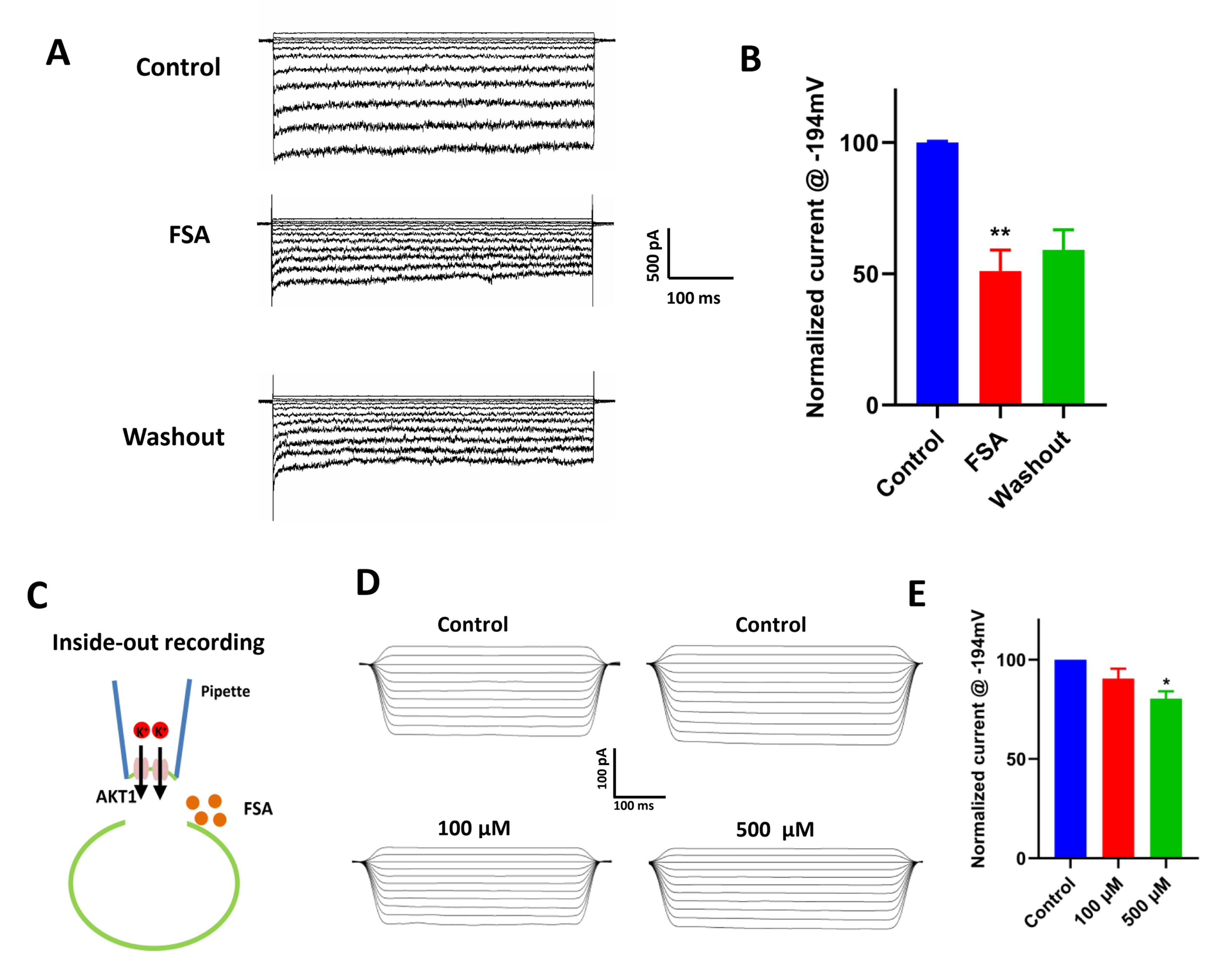

Supplement: S5 Fig — (A and B) Representative whole-cell current traces and summary showing the effect of FSA and washout on MaAKT1 currents. Data are shown as mean ± SE (n = 4), **p < 0.01 compared with control. Data are shown as mean ± SE (n = 4), **p < 0.01 compared with control (one-way ANOVA followed by Newman-Keul’s test). (C) Diagram illustrating the inside-out recording in giant patches excised from the cell membrane of HEK-293 cells overexpressing MaAKT1 channels, with FSA applied directly to the intracellular membrane surface via a pipette. (D) Representative macroscopic MaAKT1 current traces in giant patches excised from MaAKT1-overexpressed HEK-293 cells before and after 100 μM and 500 μM FSA. (E) Normalized current at -194 mV after treatment with 100 μM and 500 μM FSA in giant patches. Data are shown as mean ± SE (n = 5 for each concentration), *p < 0.05 compared with control (one-way ANOVA followed by Newman-Keul’s test). (TIF) [file ppat.1013066.s005.tif]

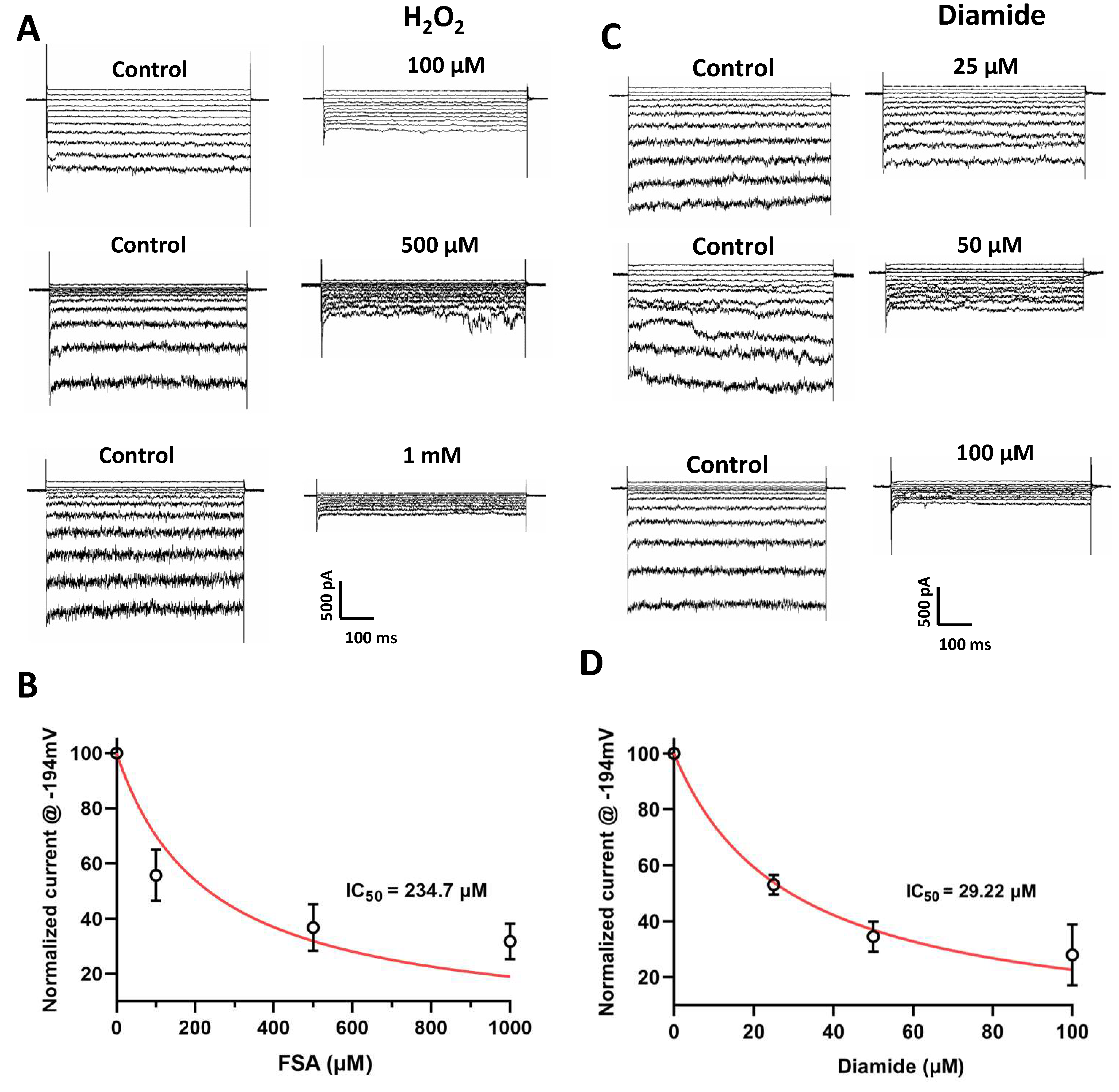

Supplement: S6 Fig — (A) Representative whole-cell current traces recorded before (left) and after (right) application of different concentrations of H2O2. (B) Normalized currents at −194 mV showing dose-dependent inhibition of MaAKT1 current by H2O2 with an IC50 of 234.7 μM (n = 3–7). (C) Representative current traces recorded before (left) and after (right) application of different concentrations of diamide. (D) Normalized currents at −194 mV showing dose-dependent inhibition of MaAKT1 current by diamide, with an IC50 of 29.22 μM. Data are shown as mean ± SE (n = 4–7). (TIF) [file ppat.1013066.s006.tif]

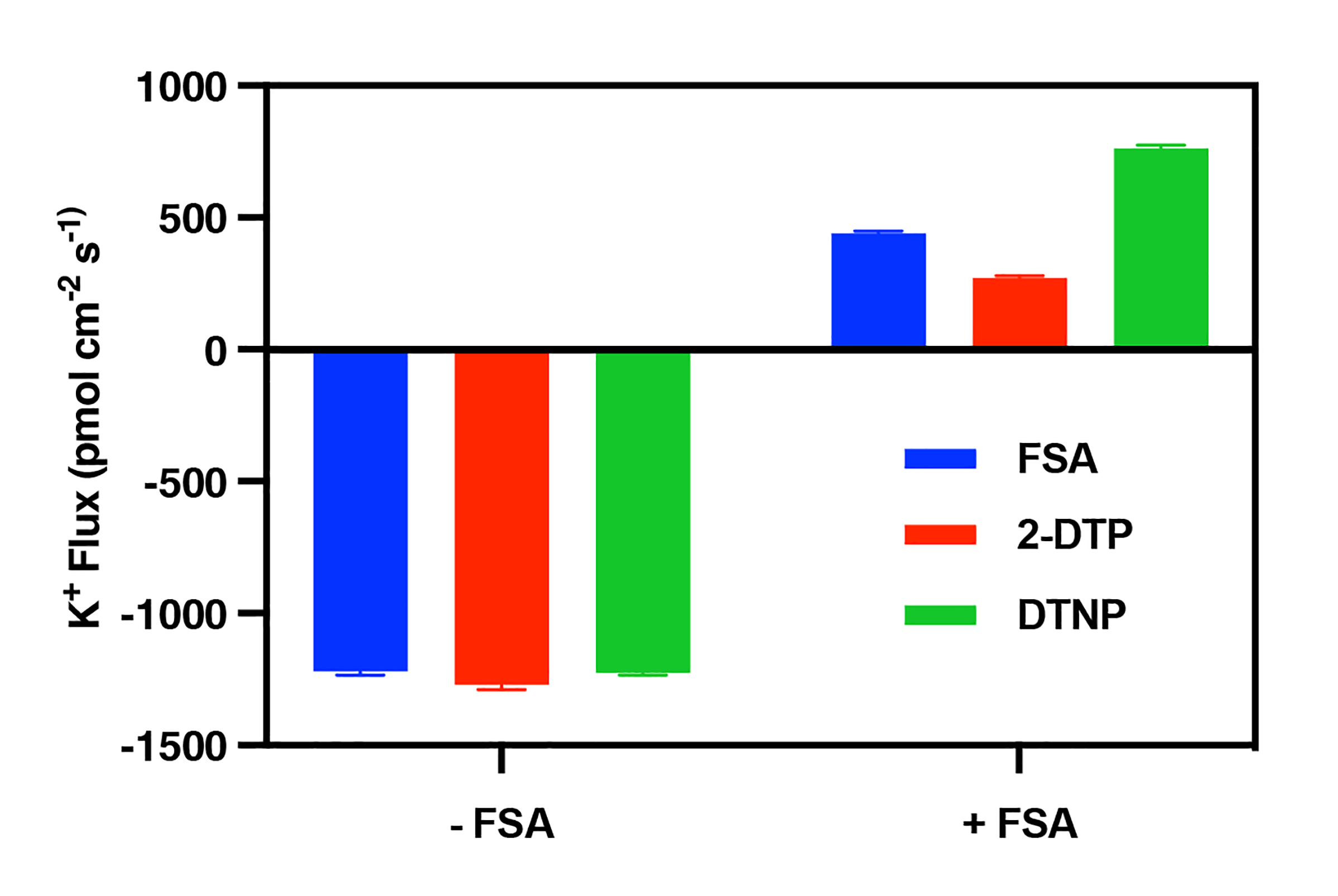

Supplement: S7 Fig — Net K⁺ fluxes were measured from banana root tips treated with FSA (20 μM), 2-DTP (50 μM), or DTNP (50 μM) using non-invasive micro-test technology (NMT). Data are shown as means ± SE (n = 6). Negative values indicate K⁺ efflux from root cells. (TIF) [file ppat.1013066.s007.tif]

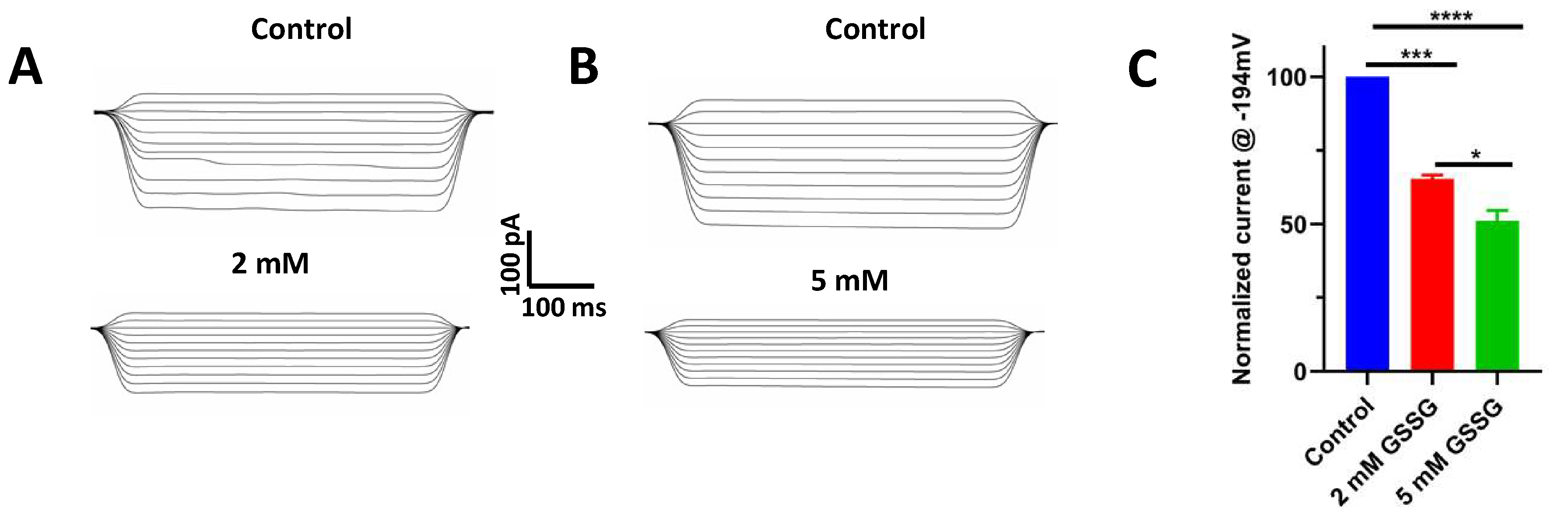

Supplement: S8 Fig — (A and B) Representative macroscopic current traces recorded in giant inside-out patches before and after the application of 2 mM (A, n = 5) and 5 mM GSSG (B, n = 9). (C) Normalized current at -194 mV showing the effect of different doses of GSSG on MaAKT1 current. Data are shown as mean ± SE, *p < 0.05, ***p < 0.001, and ****p < 0.0001(one-way ANOVA followed by Newman-Keul’s test). (TIF) [file ppat.1013066.s008.tif]

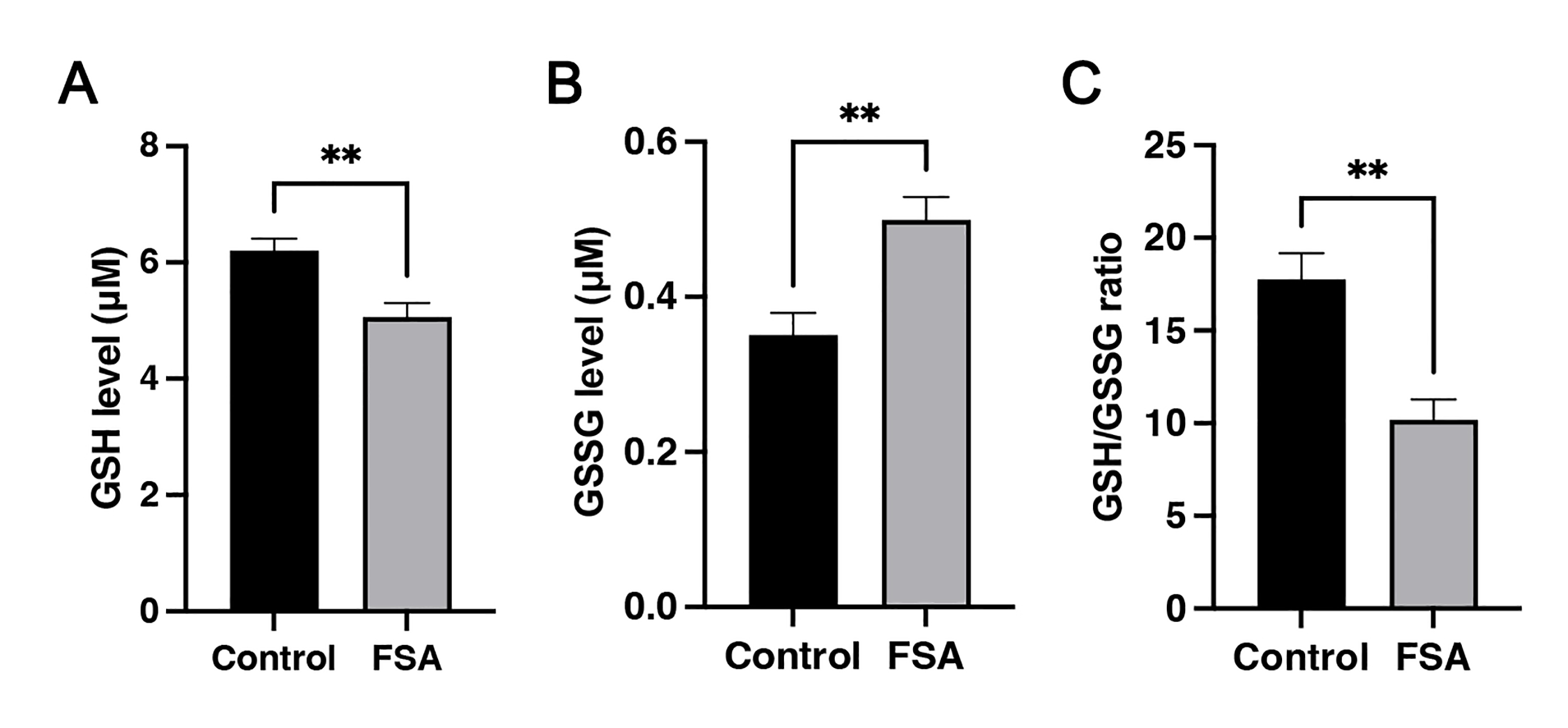

Supplement: S9 Fig — (A) GSH levels, (B) GSSG levels, and (C) GSH/GSSG ratio in the roots of banana seedlings treated with 20 μM FSA for 7 days. Data are presented as means ± SE (n = 6). **p < 0.01, Student’s t-test. (TIF) [file ppat.1013066.s009.tif]

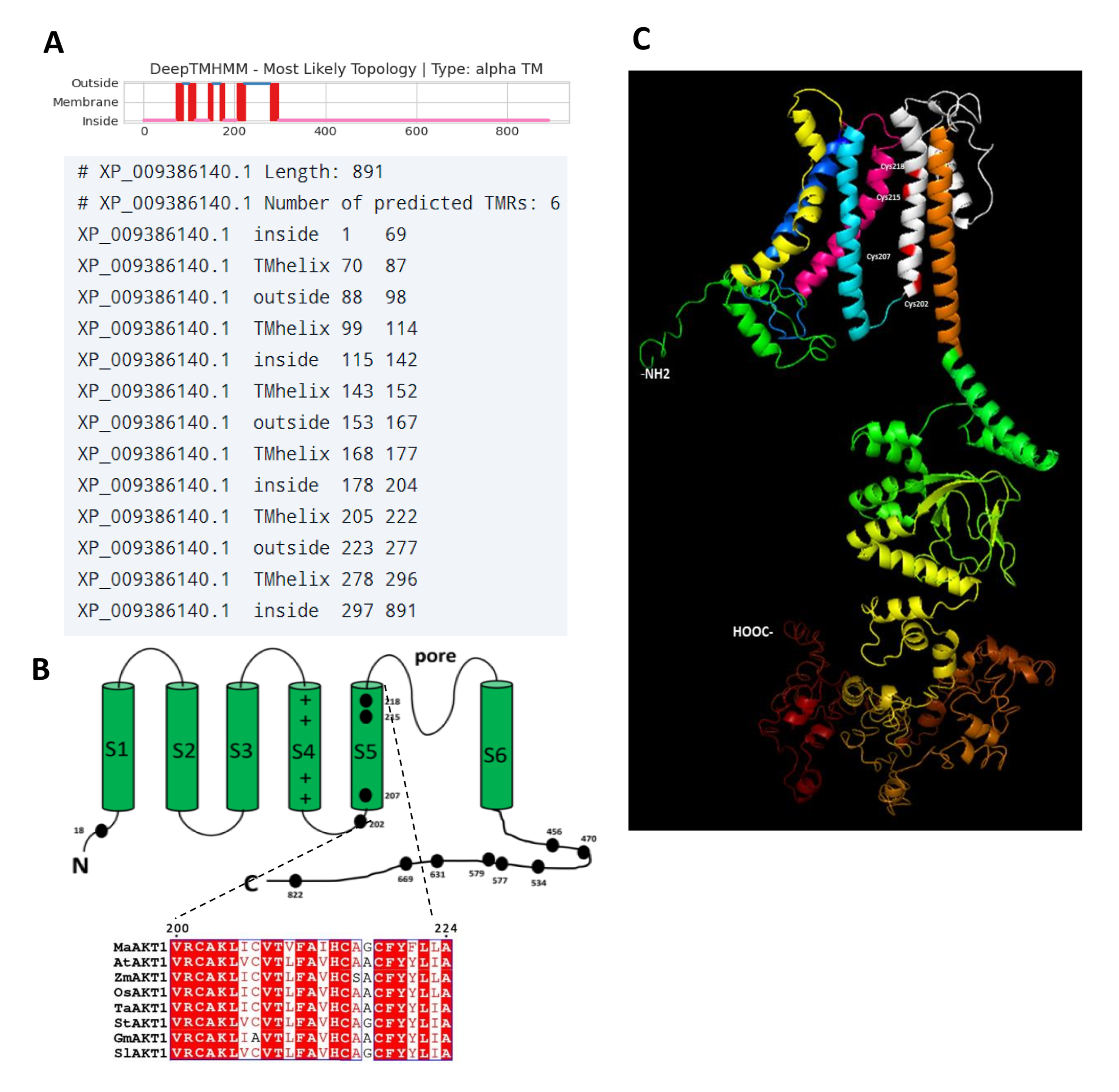

Supplement: S10 Fig — (A) Predicted transmembrane domains of MaAKT1 channel using the DeepTMHMM Model. (B) Secondary structure of the MaAKT1 channel based on predicted transmembrane domains. (C) Predicted closed-state structure of MaAKT1 by the I-TASSER protein structure prediction server using AtAKT1channel as a template. (TIF) [file ppat.1013066.s010.tif]

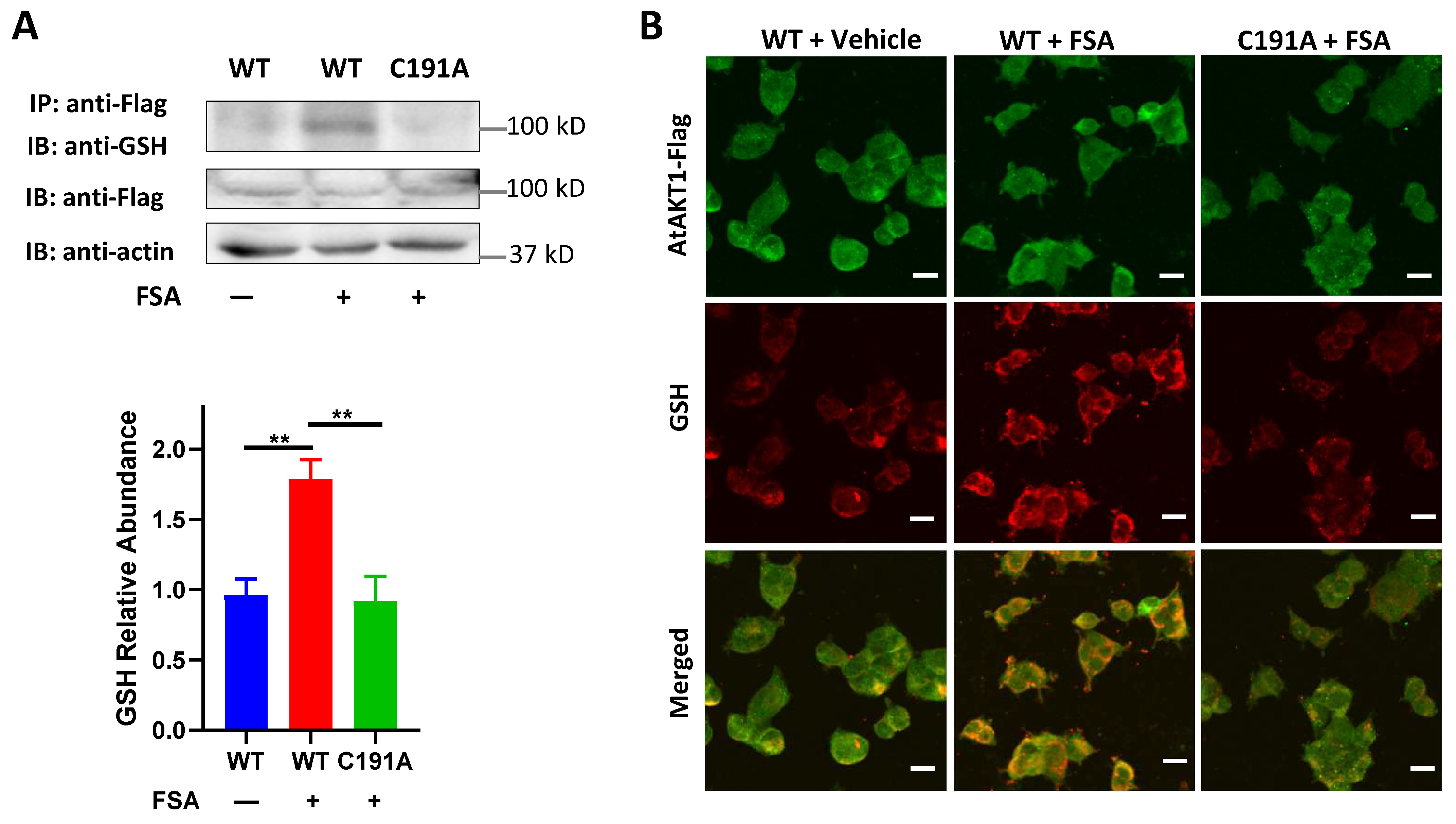

Supplement: S11 Fig — Co-IP results showing that FSA treatment promotes the biochemical interaction between GSH and AtAKT1 channels, and C191A mutation diminishes the interaction (n = 4). **p < 0.01, one-way ANOVA followed by Newman-Keul’s test. (B) Immunofluorescence images showing that FSA treatment promotes the interaction between GSH (red) and AtAKT1 channels (green) (n = 5), and C191A mutation diminishes the interaction. Bar = 20 μm. (TIF) [file ppat.1013066.s011.tif]
