# Supplementary material for: Fusaric acid-mediated S-glutathionylation of MaAKT1 channel confers the virulence of Foc TR4 to banana
Source: PLoS Pathog. 2025 Apr 9;21(4):e1013066. doi: 10.1371/journal.ppat.1013066 (PMC12040275; doi:10.1371/journal.ppat.1013066)
Supplement: S2 Fig — Sequences were aligned using MEGA 7.0. The alignment included 8 AKT1 proteins: MaAKT1 from banana (Musa acuminata, XP_009386140.1), AtAKT1 from Arabidopsis (Arabidopsis thaliana, At2g26650), ZmAKT1 from maize (Zea mays, ZEAMMB73_Zm00001d011473), OsAKT1 from rice (Oryza sativa, Os01g0648000), TaAKT1 from wheat (Triticum aestivum, CFC21_041608), StAKT1 from potato (Solanum tuberosum, NP_001275347.1), GmAKT1 from soybean (Glycine max, GLYMA_05G010600) and SlAKT1 from tomato (Solanum lycopersicum, NP_001234258.2). (PDF) [file ppat.1013066.s002.pdf]

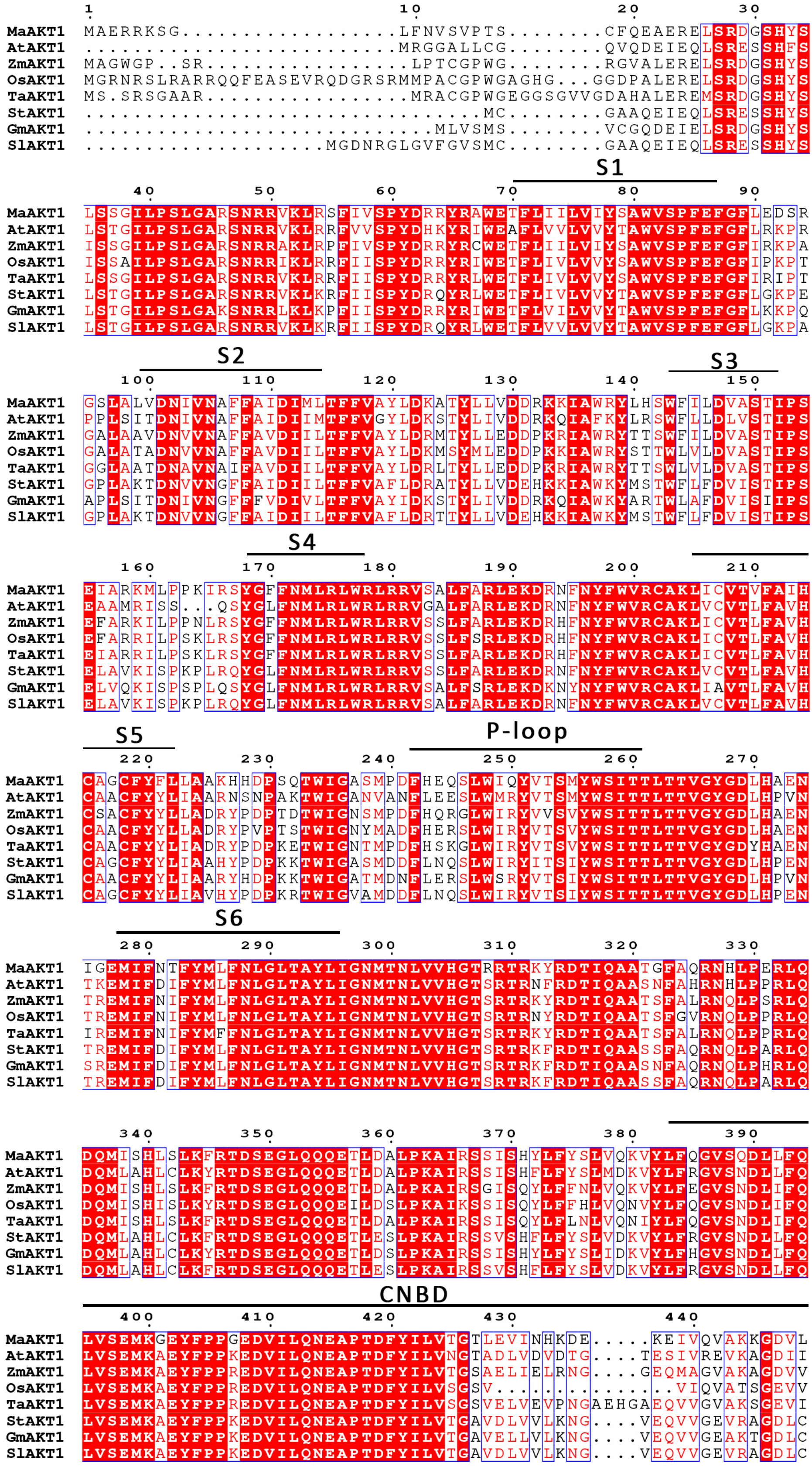
MaAKT1
AtAKT1
ZmAKT1
OsAKT1
TaAKT1
StAKT1
GmAKT1
SlAKT1
S1
S2
S3
S4
S5
P-loop
S6
CNBD

```
          450       460       470       480       490       500
MaAKT1 GEIGVLCYRPQLFTVRTRSLCQLLRLNRTVFHSIIQSNIGDGTIINNFLQHLKEQVDDP
AtAKT1 GEIGVLCYRPQLFTVRTKRLCQLLRMNRTTFLNIIQANVGDGTIIMNNLLQHLKEMNDP.
ZmAKT1 GEIGVLCYRPQLFTVRTKSLCQLLRMNRTAFLSLVQSNVADGTIIMNNLMQLLKQQKDNS
OsAKT1 GEIGVLCYRPQLFTVRTRSLCQLLRLNRTAFLSIVQSNVGDGTIIMNNLIQFLKEQKENS
TaAKT1 GEIGVLCYRPQLFTVRTRSLCQLLRMNRTAFLSIVQSNVGDGTIIMNNLIQLLKEQTDG.
StAKT1 GEIGVLCYRPQLFTVRTKRLCQLLRMNRTTFLNIVQANVGDGTIIMSNLLQHLKEMKNP.
GmAKT1 GEIGVLCYKPQLFTVRTKRLSQLLRLNRTSFLNIVQANVGDGTIIMNNLLQHLKEINDP.
SlAKT1 GEIGVLCYRPQLFTVRTKRLCQLLRMNRTTFLNIVQANVGDGTIIMNNLLQHLKEMKNP.

          510       520       530       540       550       560
MaAKT1 LMEGLLRETESMLTHGRLDLPLTLCFAVIREDDLLLRQLLKRELDPNESDNNGHTALHIA
AtAKT1 VMTNVLLEIENMLARGKMDLPLNLCFAAIREDDLLLHQLLKRGLDPNESDNNGRTPLHIA
ZmAKT1 VMVGVLKEIENMLARGRLDLPITLCFAVNKGDDLMLHQLLKRGLDPNESDNNGHTALHIA
OsAKT1 VMAGVVKEIE.................ITRGDDFLLHQLLKRGMDPNESDNDGHTALHIA
TaAKT1 VMVGVLKEIESMLARGRLDLPITLCFAVTRGDDHLLHQLLKRNLDPNESDQDGRTALHIA
StAKT1 IMEGVLLETEHMLARGRMDLPLTLCFATLRSDDLLLHHLLKRGLDPNEADNNGRSALHIA
GmAKT1 IMEGVLVDIENMLARGRMDLPVSVCFAAARGDDLLLHQLLKRGMDPNESDNNRRTALHIA
SlAKT1 IMEGVLLETEHMLARGRMDLPLTLCFATLRSDDLLLHHLLKRGLDPNESDNNGRSALHIA

                ANK
          570       580       590       600       610       620
MaAKT1 ASKGNEHCVCLLLDKGADPNRRDSEGSVPLWEAILGRHEQVAKVLRENGAQLLSGDMGLF
AtAKT1 ASKGTLNCVLLLLEYHADPNCRDAEGSVPLWEAMVEGHEKVVKVLLEHGSTIDAGDVGHF
ZmAKT1 ASKGDEQCVKLLLDYGADPNARDSEGKVPLWEALCEKHNAVIELLVESGAELSSGDTALY
OsAKT1 ASKGNEQCVRLLLEYGADPNARDSEGKVPLWEALCEKHAAVVQLLVEGGADLSSGDTGLY
TaAKT1 ASKGNEQCVKLLLEYGADPNARDSEGKVPLWEAVYAKHDTVVQLLVKGGAELSSGDTSLY
StAKT1 ASKGIENCVVLLLDFGADPNSRDSEGNVPLWEAIMGKHESVIQLLVNNGAKLSAGDVGHF
GmAKT1 ASQGKENCVSLLLDYGADPNIRDLEGNVPLWEAIVEGHESMSKLLSENGANLQCGDVGQF
SlAKT1 ASKGIENCVVLLLDFGADPNSRDSEGNVPLWEAIMGKHESVIQLLVDNGAKLSAGDVGHF

                                                ANK
          630       640       650       660       670       680
MaAKT1 ACTAAEQNSLELLEDIIRYGGDVTAAAKRDGNTALHRAVCDGNLQLVEFLLEHRADMDKP
AtAKT1 ACTAAEQGNLKLLKEIVLHGGDVTRPRAT.GTSALHTAVCEENIEMVKYLLEQGADVNKQ
ZmAKT1 ACIAVEENDAELLENVIRYGGNINNPTK.DGTTPLHRAVCDGNVQMVELLLEHGADVDKQ
OsAKT1 ACIAVEESDTELLNDIIHYGGDVNRARR.DGTTALHRAVCDGNVQMAELLLEHGADIDKQ
TaAKT1 ACTAVEQNNIELLKQILKHVIDVNRPSK.DGNIPLHRAVCDGNVEMVELLLRHGADIDKQ
StAKT1 ACVAVEQNNLSLLKEIVRYGGDVTLPKIN.GSSALHVAVCEGNIEIVKYLLDRGANVDQP
GmAKT1 ACNAVEQNSLNLLKEIMRYGGDITLPNSNTGTTALHVAVSEGNVEIVKFLLDHGASIDKP
SlAKT1 ACVAVEQNNLSLLKEIVRYGGDVTLPKIN.GSSALHVAVCEGNIEIVKYLLDRGANVDQP

          690       700       710       720       730
MaAKT1 DHQGWTPRRLADQQSHDEIKALFEGKKTSDPI........SGAPLSS......ELRRLSS
AtAKT1 DMHGWTPRDLAEQQGHEDIKALFREKLHERRV.....HIETSSSVPILKTGIRFLGRFTS
ZmAKT1 DSNGWSPRDLADQQGHDDIQALFKSRKAHRKH.......ASNGRVAP.....MLIGRFNS
OsAKT1 DGNGWTPRALAEQQGHDDIQLLFRSRKAATASGHHHVPSSTTTRVAPAAAAASLIGRFNS
TaAKT1 DSNGWTPRALAEQQGHEEIQNIFRSVIAPRKY.......TSNGRMTP.....TLLGRFSS
StAKT1 DEHNWTPRDLAEQQGHEDIKELFESRVMMR........TRSVDPIPEERG.VRFLGRFKS
GmAKT1 DKHGWTPRDLADQQAHTEIKALFDSTGEPK........VQSSFAIPERNSKIRYLGRFTS
SlAKT1 DEHNWTPRDLAEQQGHEDIKELFESRVMMR........TRSVDPIPEERG.VRFLGRFKS

           740       750       760       770       780       790
MaAKT1 EPIVPIVADAIRPPSQDGPPEKSERARRAN.FHNSLFGIISTANFGRTQGHSGMLSSVAG
AtAKT1 EPNIRPASREVSFR.....IRETRARRKTNNFDNSLFGIL.ANQSVPKNGLATVDEGR..
ZmAKT1 EPSMPDMSHENAR.VRSEVVPQKLLRKRVS.FQNSLFGVISSSHAHQDTGRLLSKG.LAG
OsAKT1 EPMMKNMIHEDAD.LPSRVLPEKLRRKRVT.FQNSLFGVISSSQAQRETDHPLSRGGLAA
TaAKT1 DPSMQKVIREDAEQQPSKVLPQ...RRKVS.FHNSLFGVISSSHPRRETDHLLSRG.LAA
StAKT1 EPTITPASHGVSFLGLDGSLGRSRPRRRSNNFHNSLFGIMSAKQTNEHDVLLSANDSN..
GmAKT1 EPTMPLPLDGSFHESQSQSQSQSRPRRRSNNYHNSLFGIMSAVHNGEKDLLSAVDMNNNA
SlAKT1 EPTISPASHGVSFLGLDGSLGRSRPRRRSNNFHNSLFGIMSAKQTNEHDVLLSANDTN..

              800       810       820       830       840       850
MaAKT1 PP...RPMLGGSGRGRGNQQRMNLLRVTISCPERG.EAADKLVLLPDSLQELLDIGSKKF
AtAKT1 ............TGNPV........RVTISCAEKD.DIAGKLVLLPGSFKELLELGSNKF
ZmAKT1 TE...GPN...CRHDS.......LIRVTISCPEKGGNAAGKLVRLPRSMAELLELGAKKF
OsAKT1 TG...SPNPSSGSRNA.......VIRVTISCPEKG.NTAGKLVLLPQTLDMLLELGAKKF
TaAKT1 TG...GPSYPQAHHNP.......LVRVTISCPEKG.NTAGKLVILPGSMKELLQLGAKKF
StAKT1 ......VSVMTTKTYAP........RVTVCCPEKG.DNGGKLILLPQSFKELLQIGSSRY
GmAKT1 RNGMKSSSASSSALGPT........RVIISCPEKG.EVVGKLVLLPGSFQELVEIGAKKF
SlAKT1 ......VSITTTKTYAP........RVIVCCPEKG.DNGGKLILLPQSFKELLQIGSSRY

                             KHA
             860       870       880       890
MaAKT1 GFSASKVLTADG.AEIDDVKLIRDGDRLVLVGGGSVAATRHD...........
AtAKT1 GIVATKVMNKDNNAEIDDVDVIRDGDHLIFATDS...................
ZmAKT1 GIKPGKVLTVGG.AEIDEVELIRDGDHILLVSDEWVPDVEQIRP.........
OsAKT1 DFAPTKVLTVEG.AEVDEVELIRDGDHLVLVSDEWDAEKMKCKS.........
TaAKT1 DMMPTKVLTIEG.AEVDEVELIRDGDHLVLASDDWIPDDTQIGGKN.......
StAKT1 GISQAKVVSKDG.AEIDEIELIRDGDRLVFVSDK...DNNIDEAKSSEL....
GmAKT1 GFYPNKVVCKDG.GEIEDLEVIRDGDHLVFLGASGVLESNCPAPAPLTNGVHI
SlAKT1 GISQAKVVSKDG.AEIDEIELIRDGDRLVFVRDK...ENNIDEAKSS......
```
